# Circadian alignment of early onset caloric restriction promotes longevity in male C57BL/6J mice

**DOI:** 10.1126/science.abk0297

**Published:** 2022-05-05

**Authors:** Victoria Acosta-Rodríguez, Filipa Rijo-Ferreira, Mariko Izumo, Pin Xu, Mary Wight-Carter, Carla B. Green, Joseph S. Takahashi

**Affiliations:** 1 Department of Neuroscience, Peter O’Donnell Jr. Brain Institute, University of Texas Southwestern Medical Center; Dallas, TX, 75390, USA;; 2 Howard Hughes Medical Institute, University of Texas Southwestern Medical Center; Dallas, TX, 75390, USA;; 3 Animal Resources Center, University of Texas Southwestern Medical Center; Dallas, TX, 75390, USA.

## Abstract

Caloric restriction (CR) prolongs lifespan, yet the mechanisms by which it does so remain poorly understood. Under CR, mice self-impose chronic cycles of 2-hour-feeding and 22-hour-fasting, raising the question whether calories, fasting, or time of day are causal. We show that 30%-CR is sufficient to extend lifespan 10%; however, a daily fasting interval and circadian-alignment of feeding act together to extend lifespan 35% in male C57BL/6J mice. These effects are independent of body weight. Aging induces widespread increases in gene expression associated with inflammation and decreases in expression of genes encoding components of metabolic pathways in liver from *ad lib* fed mice. CR at night ameliorates these aging-related changes. Thus, circadian interventions promote longevity and provide a perspective to further explore mechanisms of aging.

## One-Sentence Summary

Timed caloric restriction at night enhances longevity.

Caloric restriction (CR) without malnutrition or starvation, achieved by reducing ~30% of daily food intake, is the most effective non-pharmacological intervention that improves lifespan in model organisms (1); however, the underlying mechanisms remain unclear (2–6). Classical CR protocols in mice lead to a temporal restriction of food intake with a long (>22h) fasting interval because mice consume the food as soon it becomes available (7–9). Timed food administration is a potent signal that entrains circadian clocks in peripheral tissues such as liver (10–12). Thus, in addition to reducing daily energy intake, CR resets complex circadian programs of gene expression in tissues throughout the body (13–15). Although decreased energy intake is commonly thought to be the critical factor that extends lifespan, it is possible that the timing of food intake is a key component. The changes caused by time-restricted feeding can have profound effects on physiology (16). For example, mice (which are nocturnal) fed a high-fat diet only during the day gain significantly more weight than mice fed the same diet only during the night phase (17). Also, mice fed a high-fat diet restricted to an 8-hour window during the night were protected against diet-induced obesity, hepatic steatosis, hyperinsulinemia, and inflammation compared to mice fed *ad libitum* (AL) (18, 19). Thus, temporally restricted feeding at night, which is the normal active and feeding time of day for mice, is beneficial.

**Fig. 1.**
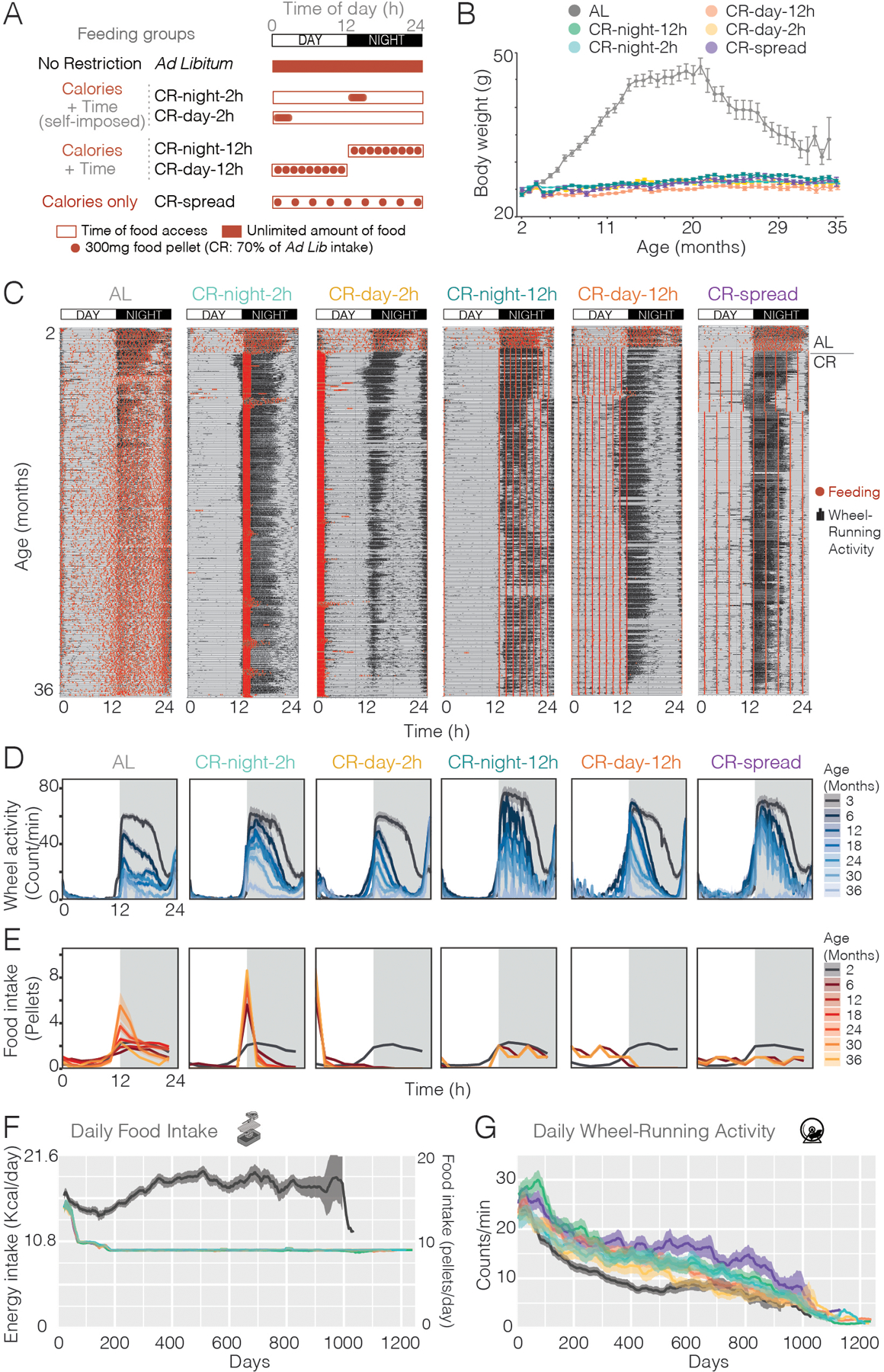
Effects of CR on body weight, circadian behavior and feeding in C57BL/6J male mice. **(A)** Experimental design showing feeding conditions for each of the six groups. **(B)** Average body weights (± SE) of mice in all 6 groups (n = 43 for AL and n = 36 for each of the CR) taken every 3 weeks throughout the experiment. **(C)** Examples of double-plotted actograms from each experimental group overlaying wheel-running (black histograms) and feeding (red dots) behaviors. All mice were on *ad lib* feeding for the first six weeks (period above the line on the right of the actograms) before the CR began. **(D-E)** 24h profile of the wheel-running activity (D) and food intake (E) at different ages (averaged over 21 days, n=36–43 mice) for each group. **(F)** Energy intake per day (left axis) and number of food pellets per day (right axis) for each group throughout the experiment. For AL group, dark line is average, gray shading is SE. All CR groups were limited to 70% of *ad lib* consumption for the first 200 days of age and was not adjusted after 200 days so no variation is observed. **(G)** Daily wheel-running activity (average counts/min over 24 hours ± SE) throughout the experiment.

Although the timing of food intake can have an impact on health, it remains unclear whether the timing and frequency of feeding also affect lifespan in mice (16, 20, 21). Food consumption triggers behavioral and metabolic changes in mammals that have profound impacts on health status (22). We dissected the contributions of feeding time and fasting under caloric restriction and compared behavioral, metabolic, and molecular outcomes throughout lifespan. We tested five different CR protocols as well as an AL control group using automated feeders (7). After 6 weeks of baseline AL food access, C57BL/6J male mice were subjected to 30% CR. Mice were fed (9–10 300-mg food pellets; 9.72–10.8 kcal) every 24h starting at the beginning of the day (CR-day) or night (CR-night) similar to classical protocols in which mice consume their food within 2h as one meal (7). In order to prevent the 2h-binge eating pattern and to reduce the fasting interval to ~12h, two additional CR groups of mice were fed a single 300-mg pellet (1.08 kcal) delivered every 90 min to distribute the food access over a 12h-window either during the day (CR-day-12h) or during night (CR-night-12h). A fifth CR group of mice was fed a single 300-mg pellet every 160 min continuously spread out over 24h (CR-spread) to abolish the rhythmic pattern of food intake and to prevent any fasting intervals (Fig. 1A).

## Behavioral and body weight dynamics with age

To select the diet for the longevity studies, we first compared standard lab chow (Teklad Global 2018) with two different precision food pellets with similar caloric content but different compositions: a grain-based diet that we used previously (F0170) (7) and a purified diet (F0075) (fig. S1A). We found that mice fed the purified diet showed similar body weight gain to the standard lab chow; however, the mice fed grain-based pellets gained significantly more weight (fig. S1B). Because the composition of grain-based diets is known to vary by batch and by season of the year (23), and because longevity experiments require at least 4 years duration, we chose the purified diet that can be completely defined and maintained over the entire duration of the lifespan experiments, and did not cause excessive weight gain compared to lab chow. We used an automated feeding system (7) and monitored feeding and wheel-running activity of individually housed mice continuously throughout their lifespan. This allowed us to measure behavioral and metabolic changes in mice under all 6 feeding conditions as they aged. In agreement with previous studies (24), mice under unrestricted feeding (AL) gradually increased their body weight until 20 months of age, after which they showed an age-related decline (Fig. 1B, and fig. S2). All CR groups maintained lower body weights throughout lifespan, consistent with lower food intake (Fig. 1B, and fig. S3). We previously showed that CR-day mice gained more weight than CR-night mice with the grain-based diet (7) but this effect was not reproduced with the purified diet (fig. S2), perhaps due to the difference in fat source of the two diets (fig. S1A). Long-term recordings of feeding events showed that mice adjusted their feeding patterns to match the externally controlled availability of food (including daytime feeding and 24h spread-out feeding). These feeding patterns were consistently maintained throughout their lifespan (Fig.1, C and E, and fig. S4A, and Data S1). Mice in the AL group normally consumed about 75% of their food at night and maintained this pattern of food consumption throughout life with a gradual increase in food consumption with age after the first year (Fig. 1F, and fig. S4A). Mice in the CR-night-2h and CR-day-2h groups with 24h access to 30% caloric restriction (relative to AL controls for the first 200 days of study) rapidly consumed their daily allotment within 2h as previously described (Fig.1, C and E) (7), and this 2-hour intake pattern was maintained throughout their lifespan (fig. S4A). Although AL mice increased their food consumption after 1 year of age, we did not increase the amount of food for the CR groups (fig. S3), and therefore the caloric restriction increased from 30 to ~40% compared to AL at later ages. Similarly, animals exposed to CR with food access spread over 12 hours or 24 hours also adapted to the imposed meal pattern, by eating each pellet as soon as it became available, which was every 90 min (CR-day-12h and CR-night-12h) or every 160 min (CR-spread) (Fig. 1, C and E, and fig. S3 and S4A). When examining the median phase of feeding, which is the time at which mice ate 50% of their daily allotment, we observed that the phase for classic CRs is 1h after the food onset (ZT 1h for CR-day-2h, and ZT 13h for the CR-night-2h). For the CR-spread, the phase is ZT12, since they eat equal amounts during the day and night (fig. S4A). The feeding pattern is also consistent with daily changes in body weight (fig. S4B). With the exception of the CR-spread group, in which the food is equally distributed throughout the day, body weight changed throughout 24h with a significant increase during the feeding time (fig. S4B). This finding is more pronounced in classic CR protocols, with highest body weight gain of 3g occurring between ZT0-4 in CR-day-2h and ZT12-16 in CR-night-2h. These body weight gains are consistent with the observation that mice eat their entire allotment (2.7–3 g) as one single meal within 2h.

All groups maintained a normal nocturnal locomotor activity pattern for life with the exception of the day-fed mice which tended to have more daytime activity (Fig.1, C and D). Overall, these long-term recordings show that when food is restricted to the daytime, mice interrupt their “rest phase” to eat but maintain most activity during the nighttime (Fig.1, C and D). Therefore, feeding and locomotor activity are misaligned with daytime feeding in these animals throughout lifespan, which would be expected to lead to adverse metabolic consequences (25, 26). Activity of the mice declined as they aged (27), with AL mice having the lowest activity levels as compared to the CR groups between 6 and 18 months of age (Fig.1G, and fig. S5, Two-way ANOVA; age, p < 0.0001; feeding, p < 0.0001; interaction NS).

## Lifespan extension by CR depends on feeding time

**Fig. 2.**
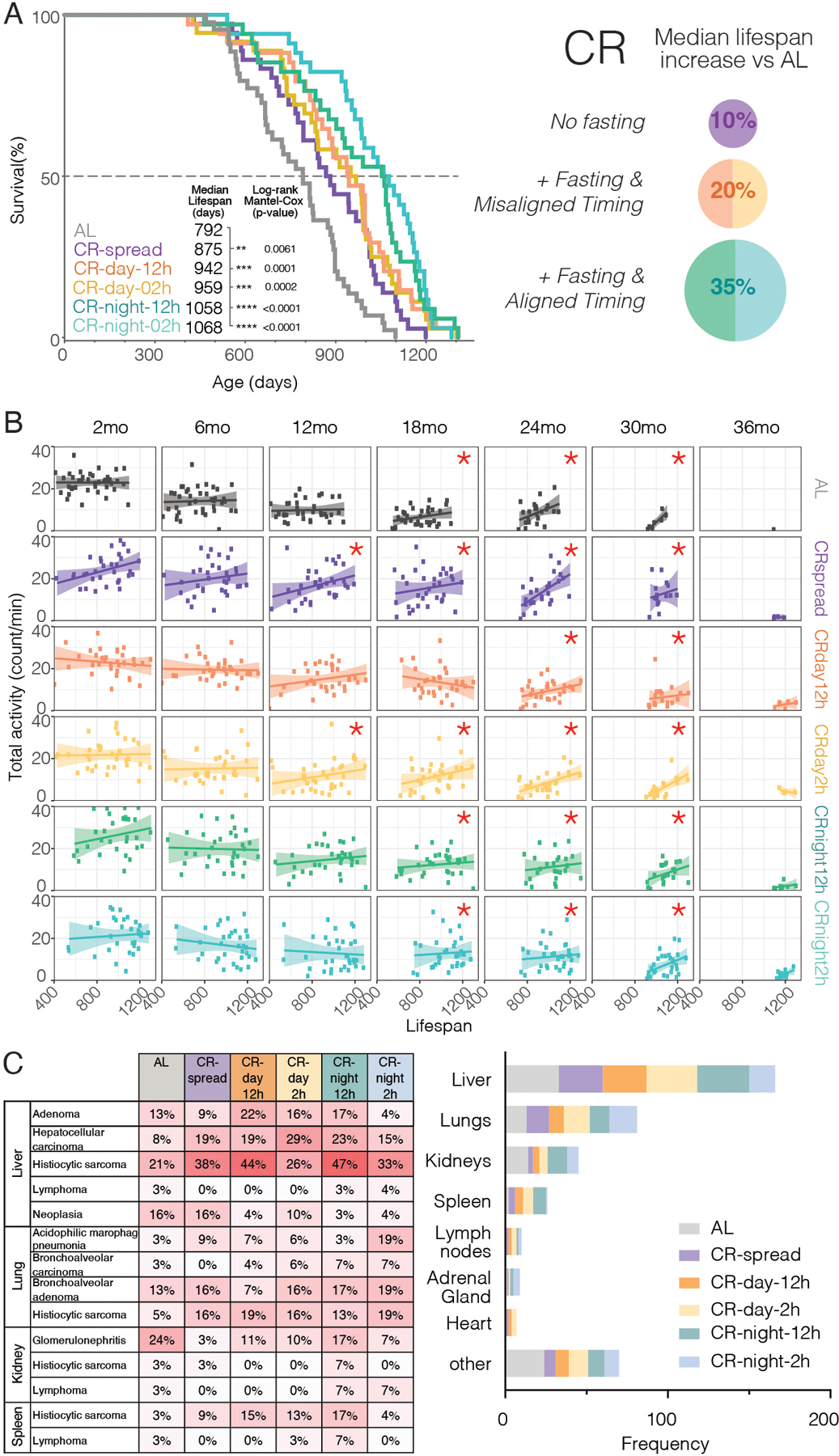
Extent of CR-mediated increases in longevity depend on feeding time. **(A)** Survival curves for each group (n = 43 for AL and n = 36 for each of the CR) are shown in left panel with median lifespan (days) inset. Right panel summarizes the results, showing the increase in lifespan from timed feeding with the largest increase when food is restricted to night. **(B)** Correlation plots comparing lifespan (days) for each mouse with its daily averaged total activity (counts/min) at different ages. Increased activity significantly correlates with longer lifespan in older (but not young) mice in all groups (see asterisks, Spearman correlation). **(C)** Necropsy followed by histopathology results showing pathologies and diseases (left) and tissues mostly affected (right) at time of death for each feeding condition.

We investigated the contribution of calories, feeding time, and fasting period on longevity. Caloric restriction was sufficient to extend median lifespan in male mice, but the range of this extension depended on when the food was consumed (Fig. 2A). The percentage of lifespan extension varied across conditions. Consistent with other reports, AL mice had a median lifespan of 792 days (24, 28). CR fed mice lived 10 to 35% longer than AL mice depending on the CR group. The CR-spread group which had a 30% reduction in calories but with feeding spread throughout the day-night cycle had a median lifespan of 875 days which is 10.5% longer than that of AL mice, demonstrating that caloric restriction alone without time restriction or fasting is sufficient to extend longevity. The CR-day-12h and CR-day-2h groups had median lifespans of 942 and 959 days, respectively, which are 18.9% and 21.1% longer than the lifespan of AL mice. Thus, in addition to the reduction in calories, a minimum of 12 hours of fasting induces its own benefits on longevity. There were no significant differences in lifespan when day fed mice fasted for ~22 hours vs. 12 hours, indicating that 12 hours of fasting is sufficient. CR-night fed mice outlived both CR-day groups: the CR-night-12h and CR-night-2h mice had median lifespans of 1058 and 1068 days, respectively, which are 33.6% and 34.8% longer than the lifespan of AL mice. Again, there was no additional benefit of ~22 hours of fasting as compared to 12 hours of fasting in these groups fed at night indicating that 12 hours of fasting is sufficient for prolonging lifespan. There was a significant extension of lifespan by CR-night-2h (34.8% extension) over CR-day-2h (21.1% extension) (Log-rank Mantel-Cox, p < 0.05), which differ in the relative phase of food consumption by the mice. It is possible that sleep disruption, because of misaligned feeding, could contribute to the difference in lifespan, and further studies are required to determine whether sleep is affected or not and if so, whether potential sleep disruptions can contribute to the differences in lifespan observed between CR-day vs. CR-night. However, it has recently been shown that sleep homeostasis is maintained in mice under a restricted feeding schedule in which food is only available for 4h in the middle of the “sleep” phase (ZT4-8) (29). In all five of the CR groups in this study, the mice consumed exactly the same number of daily calories throughout their lifespan (fig. S3–S4, Two-way ANOVA; age, p < 0.0001; feeding, p < 0.0001; interaction p < 0.0001); yet the pattern and circadian phase of feeding had major effects on lifespan. A >12-hour fasting interval combined with nocturnal (normal) feeding yielded the greatest benefits on lifespan. Thus, calories are processed differently depending on when they are consumed and anti-aging interventions, such as CR, can be optimized by timing them to a specific time of day. Maximum lifespan, estimated as the 10% longest lived mice in each group, was significantly longer in all the CR groups compared to AL, except for CR-spread (exact Fischer’s test, p < 0.05) (Data S1). Among the CR groups, only CR-night had a significant increase in maximum lifespan compared to CR-spread (exact Fischer’s test, p =0.0256 CR-night vs. CR-spread). This suggests that feeding and fasting cycles that are in sync with internal circadian clocks (CR-night-2h) extend both median and maximum lifespan, which is indicative of delaying the aging process as opposed to delaying the onset of a single disease.

Necropsy followed by histopathology revealed all groups had similar diseases at death, but in CR groups, these occurred at older ages (coinciding with longer lifespans) as previously reported (8). Neoplasias were the most frequent pathology in all groups, with histiocytic sarcomas being the first cause of death followed by hepatocellular carcinoma (Fig. 2C and Data S1). Histopathology analysis of the target tissues also revealed the highest incidence of lesions in the liver (Fig. 2C).

## Age-related decline in activity predicts lower survival in mice

To evaluate whether behavioral or metabolic parameters correlated with longer lifespans, we compared feeding (total daily intake), body weight, and wheel-running activity (total daily activity and % nighttime activity) with lifespan. We found no correlation of lifespan with food intake nor body weight at any age (fig. S6 and S7). However, in all feeding conditions, daily locomotor activity level positively correlated with longer lifespan after 18 months of age (Fig. 2B). Additionally, those mice that maintained higher activity levels during the normal circadian phase (at night) after 24 months of age also positively correlated with longer lifespan (fig. S8 and S9). Because voluntary wheel-running activity does not affect lifespan in mice (30), our results suggest that the level of wheel-running activity after 18 months of age could be a biomarker for healthspan. Thus, activity level of mice at older ages (> 18 months) can be used as a predictor of longer lifespan.

## Caloric restriction promotes widespread metabolic benefits

Body composition analysis at 12 and 20 months of age showed that while fat mass was significantly higher in AL vs. CR groups, there were no differences in fat mass among the CR groups (fig. S10). We assessed metabolic markers from plasma at 6 and 19 months of age. Insulin levels increased with age under AL, and such increases were attenuated by all CR groups, which maintained low insulin levels at both ages (fig. S11). In young mice, CR groups have similar insulin levels yet lower glucose in plasma as compared to AL (fig. S10 and S11), suggesting improved insulin sensitivity was associated with lower food intake. As the mice aged, similar levels of circulating glucose were found in all feeding groups even though AL fed mice had higher insulin levels than CR groups, suggesting that CR generally protects against age-related insulin resistance. Similar to that seen with insulin, leptin showed a significant age-related increase under AL that did not occur in the CR groups. While all CR groups maintain lower levels than AL at 19 months of age, only the CR-night groups were significantly lower at younger ages, suggesting that alignment of feeding may play a role in regulating satiety at both ages. Glucagon-like peptide 1 (GLP-1) is an intestinal hormone that is secreted after meals and decreases blood sugar levels by promoting insulin secretion and suppressing glucagon release (31). We found that although the levels of GLP-1 did not change with aging, the longest-lived (CR-night) groups had significantly lower levels at older ages as compared to AL (fig. S11). This indicates that CR-night mice may have an improved sensitivity to GLP-1 in regulating insulin secretion and glucose levels. Overall, the longest-lived CR groups had improved hormonal profiles, insulin sensitivity and glucose homeostasis as they aged.

## Gene expression changes with aging and caloric restriction

**Fig. 3.**
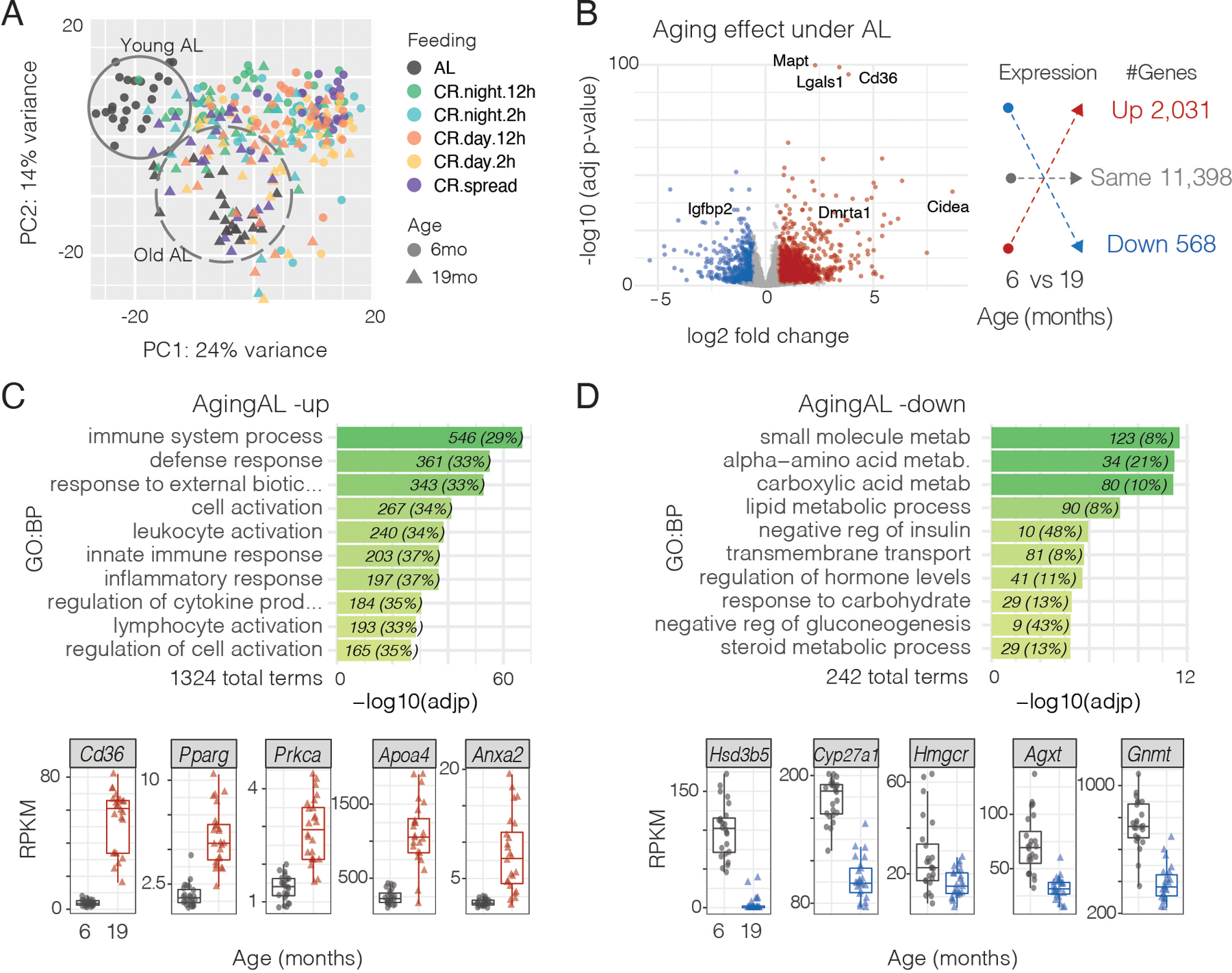
Gene expression signatures in liver change during aging in AL mice. **(A)** Principal component analysis of gene expression from liver mRNA-seq. mRNA-seq data is from 48 mice for each feeding condition (24 from 6 months of age, 24 from 19 months of age), with livers collected every 4 hours over 48 hours while mice were in constant dark. Circles indicate young AL mice (solid line) cluster together while triangle aged AL mice (dashed line) are in a distinct cluster. Liver gene expression data among CR groups cluster together independently of age. **(B)** Volcano plot showing differential gene expression in young vs. aged AL mice. Red denotes genes whose expression is significantly increased in aged AL mice; blue denotes genes that are significantly decreased in old AL mice. The number of mRNAs in each category are shown in the right panel. **(C)** Gene ontology terms of genes that are increased in aged AL mice (top) and examples of gene expression (below; gray = young AL, red = aged AL). **(D)** Gene ontology terms of genes that are decreased in aged AL mice (top) and examples of gene expression (below; gray = young AL, blue = aged AL).

To determine the effects of aging and feeding at the molecular level, we assessed circadian gene expression patterns with RNA-seq in mouse liver in all six feeding conditions at two ages (young mice at 6 months and old mice at 19 months). We chose to profile liver because it is a major metabolic target of the circadian clock system (32) and because the liver had the highest incidence of age-related lesions among target tissues (Fig. 2C). We chose 6 months of age to assess fully mature adult mice and 19 months of age to assess aged mice before their obvious decline in body weight (Fig. 1B) and before there was >10% mortality in AL mice (Fig. 2A). In each group we profiled the liver at 12 time points every 4 hours for 48 hours across two circadian cycles in mice transferred to constant darkness to assess circadian rather than diurnal cycles (2 biological replicates X 12 timepoints = 24 liver samples per feeding condition). We chose to perform these gene expression experiments in constant darkness to determine if a rhythmic gene profile is circadian. Because liver cycling gene expression patterns are strongly driven by feeding cycles (33), the absence of light:dark (LD) cycles would be expected yield similar results for rhythmic gene expression in the liver to those seen in LD cycles as reported previously (11, 33). Unbiased principal component analysis showed that: i) samples from young and old groups clustered separately under AL; ii) young groups under CR clustered together and separately from AL; and iii) old groups under CR clustered together, between aged-AL and young-CR groups (Fig. 3A). This suggests that at the molecular level, the aging process is different between mice fed AL or CR; and aged-CR mice maintain a liver gene expression pattern more similar to that of the young animals.

To address the overall impact of aging at the molecular level, we performed differential gene expression analysis between young and old mice (Data S2 and Data S3). 2599 genes (18.6% of expressed genes) were differentially expressed with aging under unrestricted AL feeding (Fig. 3B and fig. S12). Of these genes, 2031 genes were up-regulated and 568 genes were down-regulated. Gene ontology (GO) analysis revealed that the up-regulated genes are highly significantly related to immune system processes and inflammation (Fig. 3C); whereas, the downregulated genes were related to metabolism (Fig. 3D and Data S4). This is consistent with previous reports that increased inflammation and senescence are hallmarks of aging (3) and recent work on glycine-serine-threonine metabolism in longevity (34). Among the upregulated genes are *Cd36* (log_2_FC=3.8, padj=2.95 ×10^−92^), which is a multifunctional glycoprotein that acts as a receptor for ligands such as thrombospondin, fibronectin, amyloid beta, oxidized low-density lipoprotein, and long chain fatty acids among others (35); and the peroxisome proliferator-activated receptor gamma (*Pparg*, log_2_FC=1.8, padj=1.5 ×10^−28^), a transcription factor also associated with immunosenescence during aging (Fig. 3C) (36, 37). Other genes of note were: *Adora1, Serpine1, Themis,* 10 Toll-like receptor (*Tlr*) genes*, Spon2* and *Zcchc11* (Data S2 and Data S3). Among the downregulated genes, there is an enrichment in key enzymes of amino acid (*Gnmt, and Agxt*) and cholesterol metabolism such as HMG-CoA reductase (*Hmgcr*) which is implicated in liver disease and hepatocellular carcinoma (38–41) (Fig. 3D). Other metabolic genes of note were: *Got, Lepr, Lpin1, Pfkfb3, Scap, Hsd17b2, Hsd3b5,* as well as, 14 Cytochrome P450 (*Cyp*) genes and 28 solute carrier (*Slc*) genes (Data S2 and Data S3). Thus overall, there is an increase in expression of inflammatory genes and a decrease in expression of metabolic genes.

## Caloric restriction alone rescues most age-related changes observed under *Ad Lib*

**Fig. 4.**
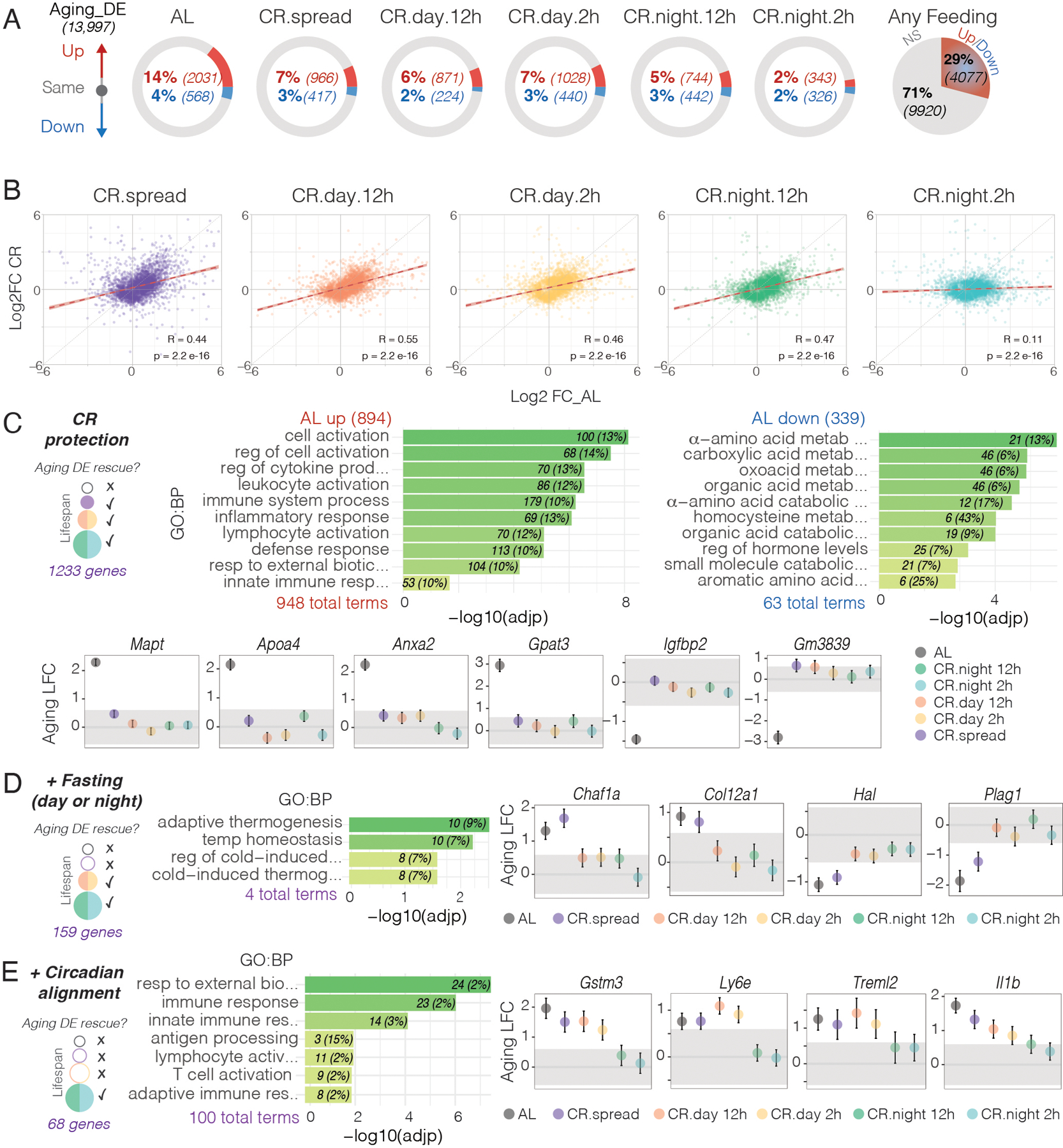
CR ameliorates age-related changes in liver gene expression observed under AL. **(A)** Schematic comparison of differential gene expression between young and old mice in the six feeding conditions. Circles show the percentage of genes that are unchanged between young and aged mice (gray), increased in aged mice (red) and decreased in aged mice (blue). Pie chart on the right shows the percentage of genes susceptible for age-related changes in any feeding condition. **(B)** Spearman correlation plots comparing changes in gene expression between the Aging DE genes between AL and CR groups. (Aging DE genes are defined here as the 4077 genes that change with age in any of the 6 feeding conditions tested). **(C)** Schematic representation of genes that are protected from age-related changes in every CR group (left) with gene ontology terms of those significantly upregulated (middle) or downregulated (right) in aged AL mice. Represented are 10 nonredundant of the top 25 most significant enriched terms. Examples of age-related fold changes (log2FC ± SE) in gene expression are shown below for all feeding conditions. Grey-shaded areas indicate FC < 1.5 considered as not significant change. Schematic representation, gene ontology and representative genes that maintain similar levels between young and old ages due to **(D)** fasting (day or night) and **(E)** circadian alignment of feeding and fasting cycles.

To determine the overall effect of caloric restriction, we analyzed which genes underwent age-related changes in any condition and identified the genes with expression levels that remain protected under CR vs AL. Across all feeding conditions there were 4,077 genes whose expression was up or downregulated with aging (Fig. 4A and Data S2), indicating that 29% of the liver transcriptome is susceptible to aging-related changes under any condition tested. AL fed mice had the highest percentage of genes that changed with aging at 18%; while all the CR groups had lower overall changes in gene expression with age (Fig. 4A). The CR-night-2h group had the smallest overall change in gene expression with age at 4% (Fig. 4A). Age-related fold change of individual genes in CR-spread and CR-day groups were partially decreased as compared to the fold changes seen in AL; whereas CR-night-2h strongly attenuated these age-related changes. Figure 4B shows these results as correlation plots of age-related fold change compared to AL for all CR groups. If there were no rescue by CR, the datapoints would fall on the unity line (slope=1); whereas, with complete rescue by CR the datapoints would fall on the horizontal line (slope=0). As seen in Fig. 4B, the regression line for the CR-night-2h group is almost completely flat demonstrating that CR-night-2h strongly reduced age-dependent changes in gene expression. Approximately 50% of age-related changes in gene expression under AL (1,233 genes) were restored in every CR condition (Fig. 4C, Data S2), by rescuing 44% of up-regulated genes (inflammation and immune function) and 60% of downregulated genes (metabolic pathways). This result indicates that CR alone prevents most of the age-related changes observed in the control AL condition.

GO analysis showed that the genes protected by CR were also associated with immune function, inflammation and metabolism (Fig. 4C, Data S4) (34). Among these genes were those encoding for microtubule-associated protein tau (*Mapt*) and apolipoprotein A-IV (*Apoa4*) whose expression is upregulated in old-AL mice but was maintained at lower (young) expression levels in all CR groups (Fig. 4C). These genes have been linked with aging and neurodegeneration in Alzheimer’s disease (3, 42). A similar aged-related upregulation occurred in the liver and was reduced by CR. Of the metabolic genes that decline with age under AL, such as insulin-like growth factor-binding protein 2 (*Igfbp2*), CR strongly attenuated *Igfbp2* changes in expression in all 5 CR groups.

### Fasting-related genes.

To evaluate gene expression changes due to fasting, we compared genes that were differentially expressed only in AL and CR-spread but remain constant in all the other 4 CR groups with some degree of fasting. We found 159 genes of this type that could be potential candidates accounting for the change from 10 to 20% lifespan extension (Fig. 4D and Data S2). Among these, *Col12a1* (Collagen Type XII Alpha 1 Chain) associated with cancer (43) which is elevated in liver fibrosis, *Plag1* (Pleomorphic adenoma gene 1) an oncogene associated with hepatoblastoma and age-related decrease in skeletal muscle (44, 45). Other genes include *Chaf1a* (chromatin assembly factor 1, subunit A) (46) and *Hal* (Histidine ammonia-lyase) involved in amino acid metabolism. Gene ontology analysis revealed that these fasting-related genes were also enriched for thermogenesis pathways.

### Time-related genes.

To evaluate the beneficial effect of feeding time, we searched for genes that maintain similar levels at young and old ages only in the CR-night fed groups with the longest lifespans (but were differentially expressed in AL, CR-spread and CR-day fed groups). We found 68 genes that were specifically protected by CR-night groups (Fig. 4E and Data S2). Gene ontology analysis revealed specific subsets of genes involved in the immune system and inflammation, such as *Gstm3* (Glutathione S-Transferase Mu 3) that protects against oxidative stress, *Ly6e* (Lymphocyte Antigen 6 Family Member E) that regulates T-cell proliferation, differentiation and activation, *Treml2* (Triggering Receptor Expressed On Myeloid Cells Like 2) and proinflammatory cytokines such as *Il1b* (Interleukin-1β) that are increased in the elderly (3). Thus, circadian alignment of feeding time adds another level of protection of immune function and age-related inflammation beyond that seen with caloric restriction and fasting.

## Circadian cycling of gene expression with aging

**Fig. 5.**
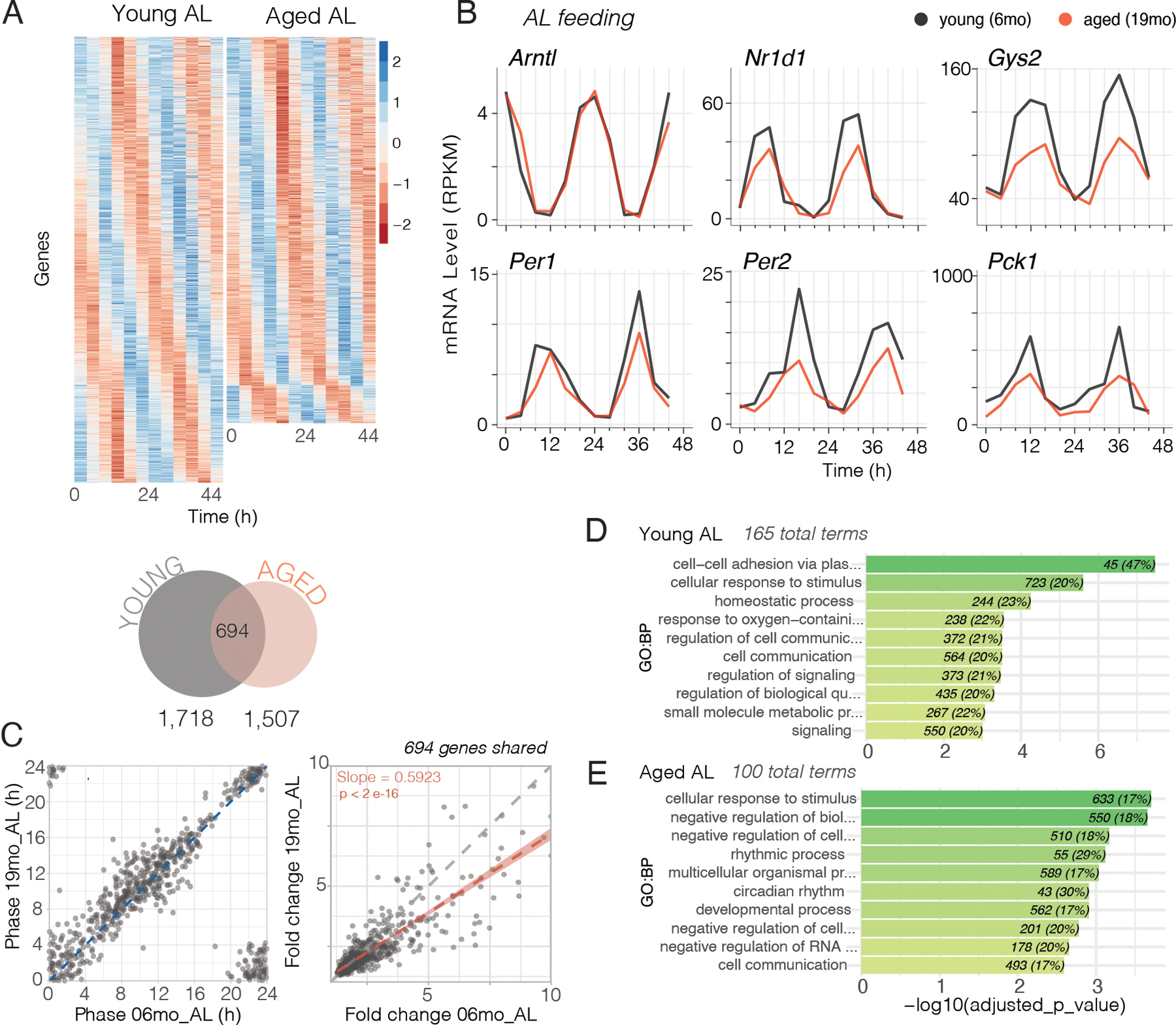
Circadian rhythms in liver gene expression are blunted during aging in AL mice. **(A)** Gene expression patterns from mRNA-seq were analyzed for circadian rhythms using ARSER, JTK_CYCLE (from Metacycle R Package) and RAIN circadian algorithms. Heatmaps (top) sorted by phase of gene expression. Each row is one gene with expression level in z-score at 12 time points (columns). Venn Diagram (bottom) shows the number of rhythmic genes in young (gray) and aged (red) AL livers using stringent criteria (significantly cycling according to three algorithms BH, p and q < 0.05 and Log_2_FC > 0.3) to define rhythmicity. **(B)** Examples of circadian profiles of genes that are rhythmic in both young and aged AL livers (black = young, red = aged). **(C)** Comparison of phase (left, hours) and amplitude (right, daily fold-change) of the 694 genes that were rhythmic in both age groups. The red correlation line (Spearman) and linear regression (slope is statistically different from 1, p < 2e-16) in the fold change comparison indicates that aged animals showed overall reduced amplitude of rhythmic genes. **(D-E)** Gene ontology terms of genes that are cycling in either (D) young or (E) aged AL mice. Represented are 10 nonredundant of the top 25 most significant enriched terms.

Robust circadian rhythms are at the core of a healthy physiology, and rhythm amplitude decreases in response to infectious diseases and aging (22, 47, 48). We investigated how feeding conditions, timing, and caloric restriction influenced circadian cycling of gene expression in young and aged mice. To search for circadian cycling genes, we performed RNA-seq from liver samples collected every 4 hours over 48 hours (Data S4 and Data S5). We used very strict criteria to identify circadian cycling genes by selecting only those genes that were significant in three out of three different algorithms (JTK_CYCLE, ARSER and RAIN) with a P value < 0.05 and a false discovery rate less than 5% (FDR < 0.05). We found 1,718 rhythmic genes in young AL mice and 1,507 rhythmic genes in old AL mice (Fig. 5A, heatmaps of cycling genes; Data S5). The overlap of these two genes sets was 694 genes. Therefore, circadian cycling genes were both lost and gained with age (Fig. 5A). Fig. 5B illustrates six cycling genes in young and old mice. Of the four circadian clock genes (*Arntl, Nr1d1, Per1, Per2*), three had a lower amplitude in old mice. Two metabolic pathway genes, *Gys2* and *Pck1*, also had lower amplitude with age consistent with the overall decline in average gene expression in metabolic pathways (Fig. 3, B and D; and see fig. S13 for example circadian profiles of pro-aging and pro-longevity genes). We compared the circadian phase and amplitude (fold change between trough and peak on the first and second circadian cycle) for the 694 shared cycling genes in young and old mice (Fig. 5A). There was no change in the phases of cycling genes with age; however, the amplitude of these cycling genes was lower as seen by the deviation of the regression line from unity (slope = 0.5941 ± 0.009, p < 0.0001) (Fig. 5C). GO analysis of these cycling gene sets showed enrichment for metabolism, cell communication, signaling, and circadian rhythms (Fig. 5, D and E and Data S3), which is consistent with the decline in average gene expression in metabolic pathways (Fig. 3D) and with analysis of circadian gene targets using ChIP-seq in which the GO category metabolism was highly significant (32).

## Effects of day vs. night caloric restriction on circadian gene expression

**Fig. 6.**
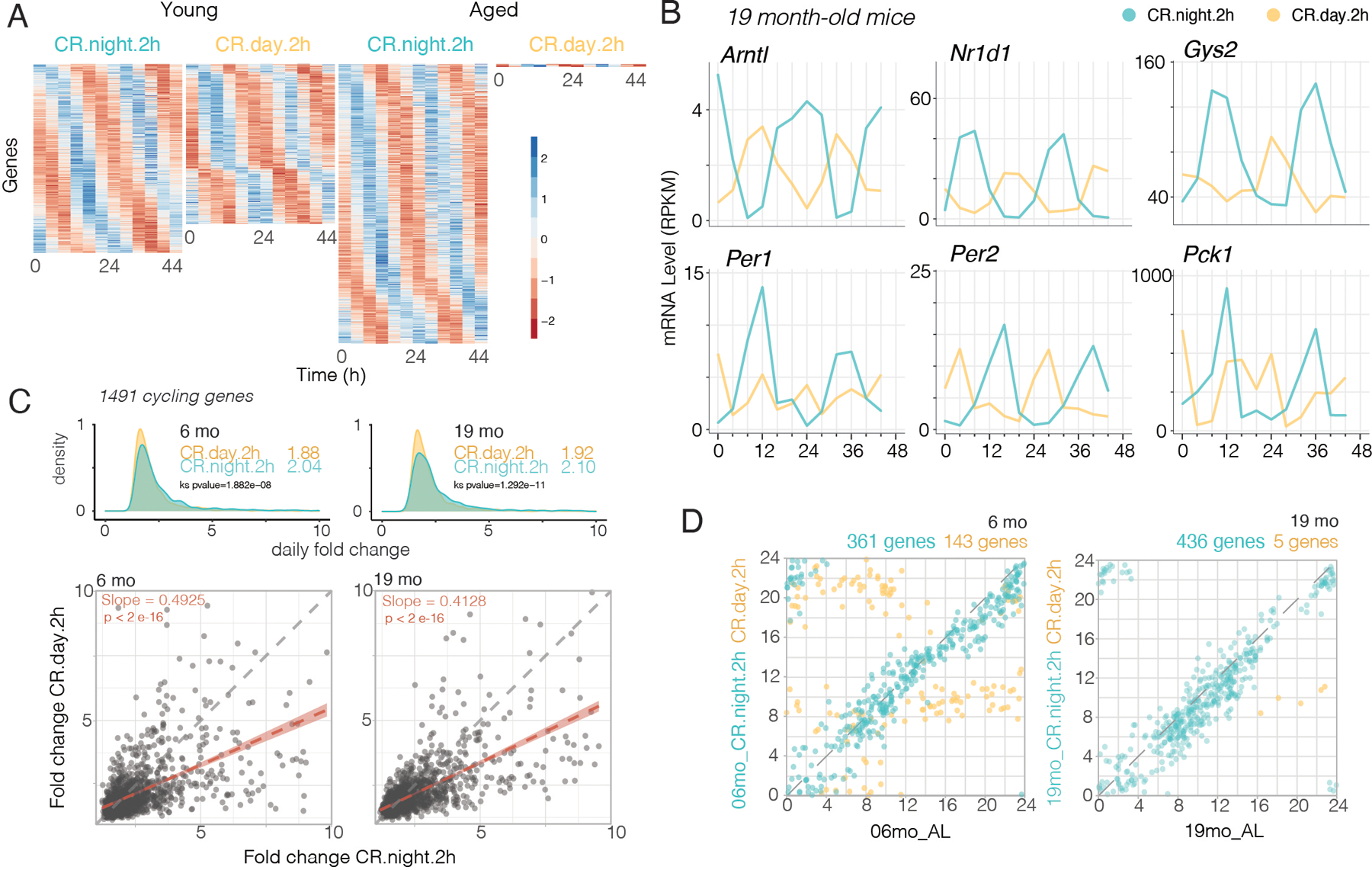
Effects of caloric restriction and phase of feeding on circadian gene expression. **(A)** Gene expression patterns from mRNA-seq were analyzed for circadian rhythms using ARSER, JTK_CYCLE (from Metacycle R Package) and RAIN circadian algorithms. Heatmaps sorted by phase of gene expression. Each row is one gene with expression level in z-score at 12 time points (columns). **(B)** Examples of the circadian profiles of the same genes shown in Fig. 5B, but comparing profiles from CR-night-2h (blue) to CR-day-2h (yellow) aged mice. **(C)** Comparison of circadian amplitude (daily fold-change) of 1491 rhythmic genes from young (left) and aged (right) CR groups. Top panels show amplitude density plots (median amplitude values are inset). Bottom panels are correlation plots comparing amplitude of genes from CR-night-2h to CR-day-2h in young (left) and aged (right) mice. The linear regression lines have slopes that are significantly less than 1 (p < 2e-16). **(D)** Phase correlation plots of rhythmic genes from young (left) and old (right) CR-night-2h-fed (blue) and CR-day-2h-fed (yellow) mice vs. AL fed mice from same ages. Phase is represented in hours. Numbers of shared cycling genes between each CR condition and AL are labeled on top of the correlation plot.

Because the median lifespan of mice in the CR-night-2h and CR-day-2h groups was significantly different (1068 vs. 959 days, respectively, and Log-rank Mantel-Cox, p < 0.05), we focused on differential gene expression in these two CR groups. One essential difference in these two groups is the phase of food consumption relative to the light-dark cycle and relative to the circadian phase of the mice as assessed by their locomotor activity rhythms. We selected the sum total genes that showed circadian cycling in at least one of these four groups: CR-day-2h and CR-night-2h from young and old mice, which led to a total of 1,491 cycling genes. Fig. 6A shows cycling genes in these four groups as a heatmap in which each line represents the color-coded levels of expression of one gene (in each row) across timepoints (columns). There was a slight reduction in cycling genes in young CR-day-2h mice, but in old CR-day-2h mice there were only 7 cycling genes. Circadian gene expression profiles of the six genes shown in Fig. 5C showed the day-fed groups at 19 months of age have either opposite phases (*Arntl, Nr1d1, Gys2, Per2, Pck1*) or disrupted circadian profiles (*Per1*) (Fig. 6B and fig. S13 for example circadian profiles of pro-aging and pro-longevity genes). We compared the fold-change amplitude of the 1,491 genes in young and old mice. At both 6 and 19 months of age, the CR-night-2h groups had a significantly higher amplitude as compared to the CR-day-2h groups as seen in the fold-change frequency histograms and correlation plots (Fig. 6C). Thus, CR-night-2h feeding enhanced circadian amplitude relative to that of CR-day-2h. To assess the phase of entrainment in these four CR groups, we compared the phases of cycling genes relative to those of AL mice at 6 months and 19 months of age. Fig. 6D shows correlation plots of genes that overlap with AL mice in either the CR-day-2h or CR-night-2h groups at 6 months and 19 months of age. The CR-night-2h mice shared many more cycling genes with AL mice than did the CR-day-2h mice at both ages. In addition, the phases of gene expression of CR-night-2h mice of both ages were correlated with the phases of the genes from the respective AL age group, with the data points falling on the unity line. As expected, the CR-day-2h mice had opposite or divergent phases relative to those of AL mice. For the old CR-day-2h mice, only 7 genes were scored as cycling and only 5 overlapped with those of AL mice. Thus, in CR-day-2h mice there is a paucity of cycling genes and this declines precipitously with age. A similar reduction was seen in fold-change gene expression in CR-spread mice compared to CR-night-2h mice at 6 months and 19 months of age (fig. S14). The CR-night-2h groups show robust circadian cycling, even in the old mice, suggesting that this intervention is very effective in rescuing circadian cycling of gene expression relative to CR-day-2h. A proviso to this cycling analysis is that these cycling algorithms (JTK_CYCLE and ARSER) are biased towards sinusoidal waveforms and although RAIN can search for spiky or sawtooth waveforms, our requirement that a cycling gene is significant (FDR<0.05) in all three algorithms results in only genes with sinusoidal waveforms passing this threshold. Daytime feeding and 2-hr feeding bouts both modify the gene expression times series waveforms to be less sinusoidal and this contributes to the lower number of cycling genes being called in the CR-day-2h group. Therefore, we do not recommend putting too much weight on the number of cycling genes called, but rather emphasize the phase and amplitude of gene expression.

## Discussion

Classic caloric restriction protocols not only reduce energy intake, but also lead to severe behavioral time-restricted feeding behavior and prolonged fasting intervals (7–9). Because time-restricted feeding and fasting are beneficial to health, these two factors may contribute to lifespan extension in classic caloric restriction experiments. In the work presented here, we deconvolved the effects of calories, fasting, and circadian alignment on longevity. We compared five different caloric restriction feeding groups that differ only in the daily pattern of food consumption without any changes in food composition and energy content. We used an automated feeding system (7) to test whether the timing and fasting period between meals impacted lifespan in male mice under 30% CR, by allowing food access only during the day, at night, or evenly distributed throughout 24h. By spreading out food throughout 24 hours, with no day-night feeding pattern in the CR-spread group, we found only a ~10% extension of median lifespan compared to AL fed mice. The CR-day fed groups that had a ~12- or ~22-hour fasting interval lived ~20% longer than AL control mice. Importantly, the degree of lifespan extension was significantly longer when food was consumed during the nighttime, which is the normal feeding time in nocturnal rodents (~35% vs. 20% compared to AL log-rank Mantel-Cox p<0.0001, and ~10% night vs. day log-rank Mantel-Cox p<0.05) (Fig. 2A). While potential sleep disruption needs to be carefully studied in CR-day vs. CR-night groups, recent evidence shows that sleep homeostasis is maintained with day-time feeding (29). All CR groups maintained a relatively steady body weight throughout lifespan, indicating that the additive effects of CR combined with appropriate timing of food consumption were independent of weight gain. Thus, the maximal benefits of CR can be achieved by a fasting interval of >12 hours in which a time-restricted feeding interval occurs in phase with the natural nocturnal circadian phase of feeding in mice (i.e., circadian alignment).

These results are consistent with recent studies in C57BL/6J male mice where similar lifespan extensions were reported in once-per-day CR fed in the morning (20%, equivalent to our CR-day-2h group) (9) or ~28% CR when food was consumed during the daytime but closer to the normal active phase at night (9h later than our CR-2h-day and 3h earlier than our CR-2h-night groups) (8). Pak et al. (9) suggest that fasting alone drives the geroprotective effects of CR, which is partially consistent with our results; however, they argue by using a 50% cellulose-diluted diet that 30% CR does not extend lifespan, and they did not test whether the phase or circadian alignment of CR could be a factor. In contrast, we showed that 30% CR without dilution of the diet in the CR-spread group extended lifespan by ~10%; thus, we conclude that 30% CR alone without fasting or circadian alignment accounts for a 10% extension of lifespan. In our study the diets used were identical in all six groups, which mitigates against the confounding effects of diet composition such fiber (cellulose) and uncontrolled grain-base diets (23, 49, 50). Therefore, our conclusions differ with Pak et al. (9) in two ways: first we find that CR alone without fasting can still extend lifespan by ~10% using the same diet; and second we find that fasting contributes in an additive manner to lifespan extension rather driving the effects of CR.

In non-human primates, the effects of CR on longevity have differed between studies performed at the University of Wisconsin (UW) and the National Institute of Aging (NIA); however, many differences in study design, diet and feeding protocols have been documented, and the overall conclusions from a joint consensus is that CR improves health and survival in rhesus monkeys (51–53). CR did not extend lifespan in the NIA study; however, the “AL” control group was longer lived, slightly caloric restricted, food was provided twice a day, and body weights were lower than AL controls in the UW study. In addition, in the UW study, food was provided *ad libitum* during the day from ~8 am to ~4 pm, but food was removed during the night. Thus, in both studies the AL groups were either partially calorie restricted (NIA) or were under a time-restricted feeding protocol (UW), which should be beneficial. Thus, it would be of considerable interest to explore the effects of time-restricted feeding and circadian phase of CR in future studies in non-human primates.

Benefits of circadian alignment of feeding and fasting cycles are widespread across species. They increase lifespan in flies (54), protect against metabolic disorders in mice (17, 18, 55, 56) and promote health in humans (57, 58). We also found that CR promotes widespread metabolic benefits, which include lower body weight with reduced fat content. Furthermore, CR (particularly the longest-lived groups, CR-night) attenuated age-related changes observed under AL by improving glucose homeostasis, insulin sensitivity and hormonal profiles.

Using circadian gene profiling, we found that timing of food intake led to complex genome-wide reprogramming of circadian gene expression in the liver consistent with others (11, 14, 33, 59–61). This emphasizes the importance of considering the sampling time, which is often a snapshot, before concluding whether any intervention increases or decreases the expression of a gene of interest. If a gene has a circadian oscillation, a single snapshot could lead to opposite conclusions depending on time it was taken, particularly in classic CR protocols resembling our CR-day-2h group (1, 7).

Aging was associated with increased expression of genes involved in immune processes and inflammation and decreased expression in genes involved in metabolism and circadian biology. CR treatment restored or attenuated many of these age-related changes in gene expression similar to that reported by the de Cabo (24, 34) and Sassone-Corsi (14) groups. Decreases in circadian amplitude of gene expression with age were reduced by CR. Overall, feeding time and fasting had additive effects on CR-mediated lifespan extension. Together, CR and time restriction of feeding to the night optimally extended lifespan and delayed many of the age-related gene expression changes in immune function, inflammation and metabolism. Further studies are required to determine whether disruption of circadian sleep/wake cycles by daytime feeding also contributes to the day vs. night differential responses in the liver transcriptome and lifespan. Thus, circadian interventions such as timed feeding can enhance the well-known lifespan benefits of CR.

There are many links between the circadian system and aging (20). High-amplitude circadian rhythms correlate with well-being (13, 62, 63), whereas clock dysfunction leads to metabolic disorders, premature aging and reduced lifespan (42, 64–67). During normal aging, rhythms decrease in amplitude and often exhibit a shift in phase (14, 20, 68–72). Interestingly, in both flies and mice, the effects of dietary restriction on lifespan require core circadian clock genes (73–75). The circadian system regulates the majority of metabolic pathways implicated in longevity (3, 4, 13, 22, 32, 59, 76, 77). Molecules known to function in the regulation of lifespan by dietary restriction, such as insulin and IGF-1 (insulin-like growth factor 1), SIRT1 (Sirtuin1), NAMPT (nicotinamide phosphoribosyltransferase), AMPK (AMP-activated protein kinase), PGC-1α (PPARγ coactivator 1), mTOR (mechanistic target of rapamycin), GSK3β (glycogen synthase kinase 3β), are all intricately involved in the molecular mechanisms of circadian clocks (77–89). Importantly, the master circadian transcription factors, CLOCK and BMAL1, have direct gene targets in every fundamental metabolic pathway in the liver (25, 32, 90, 91). Because of these direct links among the pathways involved in aging and longevity, metabolism and the circadian clock, our results demonstrate the importance of timing of CR and indicate that optimizing the phase of circadian gene expression may be a powerful intervention for extending lifespan.

We used C57BL/6J male mice; however, there could be strain and sex-specific responses worth studying further (24, 92), since, for example, ovarian hormones can protect females against dietary challenges that otherwise disrupt circadian rhythms in males (55). In future work, both sexes and multiple genetic backgrounds (92–94) should be used to explore the broader effects of circadian interventions on aging and may support application of circadian-timed interventions in human studies.

## Supplementary Material

Suppl Materials

Supplemental Data table 1

Supplemental Data table 2

Supplemental Data table 3

Supplemental Data table 4

Supplemental Data table 5

Supplemental Data table 6

## Data Availability

Data are available in the main text or the supplementary materials. RNA-seq data have been deposited in GSE190939.
