# Supplementary material for: Circadian alignment of early onset caloric restriction promotes longevity in male C57BL/6J mice
Source: Science. Author manuscript; Available in PMC 2022 Jul 7. (PMC9262309; doi:10.1126/science.abk0297)
Supplement: Suppl Materials [file NIHMS1820506-supplement-Suppl_Materials.pdf]

Science
AAAS

Supplementary Materials for

Circadian alignment of early onset caloric restriction promotes longevity in male C57BL/6J mice

Victoria Acosta-Rodríguez, Filipa Rijo-Ferreira, Mariko Izumo, Pin Xu, Mary Wight-Carter, Carla B. Green, Joseph S. Takahashi

Correspondence to: carla.green@utsouthwestern.edu, joseph.takahashi@utsouthwestern.edu

This PDF file includes:

Materials and Methods
Figs. S1 to S14

Other Supplementary Materials for this manuscript include the following:

Data S1 to S6

Materials and Methods

Animals

C57BL/6J male mice (6 week-old, n = 511) were obtained from the Mouse Breeding Core, Wakeland lab, UT Southwestern Medical Center, Dallas, TX, USA. Mice were allowed to acclimatize for 2 weeks and were then individually housed in standard polycarbonate mouse cages (Fischer Scientific, Cat. Nos. 01-288-1B and 01-288-21) containing a 4.75 in diameter stainless steel running wheel, inside isolation cabinets containing 12 cages each (*95*). Temperature and humidity levels were monitored, and the mice were housed under light/dark cycles of LD12:12 h (green LEDs, ~100 lux at the level of the cage floor) (*95*). Water was provided *ad libitum* throughout the study. The Institutional Animal Care and Use Committee (IACUC) of the University of Texas Southwestern Medical Center approved the animal protocol (APN 2015-100925), which has been subsequently renewed every 3 years (in 2018 and 2021).

Feeding and wheel-running behavior

To select the food composition for long-term experiments, mice were fed *Ad Libitum* with regular chow Teklad Global 18% Protein (Envigo), and two types of precision 300-mg pellets: grain-based (F0170, Bio-Serv) and purified diet (F0075, Bio-Serv) (n=9 mice per diet) (fig. S1A). Food intake was measured by manually weighing the food remaining every 7-21 days. Wheel-running activity was monitored remotely every day, and all mice were visually inspected every week during cage change. Body weight for each animal was measured every 7-21 days.

For longevity and gene expression experiments, mice were fed with round pellets of 300 mg each containing 3.60 Kcal/g (Dustless Precision Pellets®, Rodent, Purified, F0075, BioServ, Flemington, NJ, USA) (fig. S1A). The composition of the pellets is similar to regular mouse chow (18.7% protein, 5.6% fat, 59.1% carbohydrates and 4.7% fiber). Food access was controlled by an automated feeder system designed in our lab with Phenome Technologies Inc., Skokie, IL, USA that precisely controls the duration, amount and timing of food access (*7*). After six weeks of recording under *ad libitum* food access, mice were randomly assigned to one of 6 feeding conditions: 24h access *ad libitum* (AL, n = 91 mice); 70% of baseline *ad libitum* levels fed at the beginning of the dark (CR-night) or light (CR-day) phase which they consumed in less than 2h; or fed under CR evenly spread over 12 hours during the dark (CR-night-12h) or light (CR-day-12h) by releasing one 300 mg pellet every 90 min; or evenly spread over 24 hours (CR-spread) by releasing one 300 mg pellet every 160 min. Each CR group feeding condition consisted of n = 84 mice (Fig. 1A). Every 21 days, the mice were weighed (in the morning), cages were changed, and bedding was checked for any evidence of food spillage. We used ClockLab Chamber Control Software v3.401 (Actimetrics Inc., Wilmette, IL, USA) to program and record feeding events.

Wheel-running behavior was recorded throughout the study using ClockLab Data Acquisition System v3.209 (Actimetrics Inc., Wilmette, IL, USA). All 511 mice were monitored in real-time in these conditions, throughout their lifespan (n=34-43 per feeding) or until the time points for the tissue collections (see tissue collection, n=24 per feeding per age).

Unless indicated otherwise, feeding and wheel-running activity data is represented as an average of 21 days per mouse (which is when the cage changes occur). Related to Fig. 1D-G, Fig. 2B, and fig. S3, S4A, S5, S7-S9 and Data S1.

Survival study

The feeding and wheel-running activity for each mouse was monitored remotely on a daily basis. Mice were flagged for visual inspection when either the wheel-running activity was below

10% activity (1.14 counts/min within 24h) or the daily food intake was less than 50% of their normal consumption. This allowed us to minimize unnecessary disturbances in mouse behavior. All mice were visually inspected every 10 days (when refilling the food hoppers) and weighed every 21 days during cage/water bottle change. As mice aged and become frail, health issues were visually assessed on a daily basis by our laboratory staff with an independent assessment by Animal Resource Facility (ARC) veterinary staff. The criteria for euthanasia for moribund mice was based on an independent assessment by ARC veterinarian according to AAALAC guidelines; in which the overall condition of the animal was considered incompatible with continued survival. Briefly, we adopted the criteria for imminent death described by the Jackson Lab (https://www.jax.org/research-and-faculty/research-labs/the-harrison-lab/gerontology/life-span-as-a-biomarker); including unresponsiveness to touch, failure to eat or drink, slow respiration and cold to the touch, hunched up with matted fur and signs of sudden weight loss (>20%) and body condition score of 1. Both mice euthanized or found dead were represented as deaths in the survival curves. Mice euthanized due to injuries unrelated to imminent death were censored (total 6 censored deaths, two for CR-night-12h, two for CR-night-2h and two for CR-day-12h). For survival, two cohorts of n=24-36 and n=12 mice/feeding group were started 12 months apart. Sample size was selected to detect a 10% increase in lifespan with 80% power, using mean and standard deviations from published data (*24*).

Necropsy and Histopathology at Death

All mice in the survival study were subjected to gross necropsy (n=34-43 per group). Further histopathology studies were performed for >70% of the mice per group. Brain, liver, heart, kidney, spleen, lung, and abnormal tissues were fixed in 10% neutral buffer formalin, embedded in paraffin. Hematoxylin and eosin (H&E) stained slides were prepared and reviewed by an independent pathologist (UTSW Animal Resource Center), who was blinded to experimental conditions.

Metabolic phenotyping and body composition

Levels of hormones from plasma were determined using Meso Scale Discovery (U-PLEX Metabolic Combo, K15297K) according to the manufacturer's instructions. Mouse body composition was determined using an 100H-EchoMRI Body Composition Analyzer. Each mouse was assessed twice, at lights on (ZT00h) and lights off (ZT12h), to have even and matching light:dark and feeding:fasting cycles for each group.

Glucose Measurement

Blood was collected from a small cut in the tail and was measured using the Precision Xtra blood glucose meters and glucose test strips (Abbott Diabetes Care Inc., Almeda, CA). Blood glucose levels were measured and averaged at various time points throughout the day. For the 8 month-old, we used n=4 mice/feeding with repeated measures, following the same 4 mice every 4h during 24h. For the 19 month-old, we used n=12 mice/feeding (independent measurements, 2 mice x 6 timepoints collected every 4h during 24h tissue collection).

Tissue collection

48 mice for each of the 6 feeding conditions were assigned for RNA-sequencing experiments and divided into two sets for 6 months and 19 months of age (24 mice per feeding condition). For each age group, mice were released into constant darkness and the feeding schedules were maintained. Tissue samples were collected after 36 hrs of constant darkness

beginning at timepoint CT0. Samples were collected every 4 hours for 48 hours. Tissues were snap frozen and stored at -80°C. Plasma was extracted using K2-EDTA tubes with DPP4 (Millipore) and protease inhibitors (S8830) on ice followed by centrifugation 3,000 rpm for 10 min at 4°C.

Liver transcriptomics (mRNA-sequencing)

RNA was isolated from frozen livers homogenized in TRIzol and extracted using Zymo columns (Direct-Zol RNA MicroPrep, cat. R2062) according to manufacturer instructions. All RNA samples used had a RIN >8; 260/280 ratio >1.8; and 260/230 ratio >1.8. Single-stranded mRNA-seq library preparation was performed as described previously (*96*). Briefly, libraries were prepared with 25 ng of mRNA, poly(A)-selected from 5 μg total RNA, with 13 cycles of PCR amplification. Library size was confirmed by Tapestation 2200 (Agilent) (*97*). Single-end sequencing of 75 base pairs was performed using NEXT-seq 5500 (Illumina) at the McDermott Sequencing Core at UT Southwestern. Read quality was assessed using the FASTQC quality control tool (http://www.bioinformatics.babraham.ac.uk/projects/fastqc). Reads were mapped with STAR (*98*) following Cutadapt trimming (*99*) to M14 Genecode GTF (protein coding, https://www.gencodegenes.org/mouse/release_M14.html). Reads per kilobase per million (RPKM) was used to normalize uniquely mapped reads according to the sequencing depth (an average of 10M uniquely mapped reads per sample) and gene length.

Differential gene expression analysis

Liver mRNA-seq datasets from male mice were combined (24 samples/feeding condition collected every 4 hours over 2 days). Genes with low counts were removed, and “expressed genes” were defined as those with at least 3 counts in 24 samples. 13,997 Genes passed this expression cutoff. VST was used to normalize RNA-seq across the 288 samples. PCA plots were used as an unbiased approach to visualize the distribution of clusters by age x feeding. DESeq2 (*100*) was performed to address age-related changes in overall levels of gene expression in young vs. aged (6 vs. 19 month-old), and differentially expressed genes were defined as FDR < 0.05 and fold-change cutoff $\geq$ 1.5 (greater or equal to).

Circadian expression analysis

Liver mRNA-seq datasets from male mice, with biological duplicates (24 samples/feeding conditions) were collected every 4 hours over 2 days in the dark. Expressed genes were further trimmed to 10,248 genes to include only those that pass the previously defined cutoff and whose expression is > 0.5 RPKM as an average across all conditions and ages. Three cycling algorithms were used to search for genes whose expression cycle in a circadian manner: ARSER (*101*) and JTK_CYCLE (*102*) (implemented in Metacycle R package, (*103*)) and RAIN (*104*). Genes were considered cycling if the p and q values were 0.05 or lower for all three algorithms (ARSER, JTK_CYCLE and RAIN) and the daily fold change over $Log_2(FC) > 0.3$. In addition to amplitude, all 3 algorithms used are biased by the waveform of the rhythms. Both ARSER and JTK_CYCLE favor detection of sinusoidal waveforms; whereas, RAIN allows detecting rhythms with narrow peaks or troughs (*104*). While we focus on genes that can be detected as cycling regardless of the algorithm used, we also provide the individual results of each algorithm in Supplemental Data S6 in case other researchers prefer to use different criteria or threshold cutoffs for cycling. Our strict criteria for significant cycling genes leave many genes out even if other 2 algorithms call it cycling, as we required all 3 of 3 algorithms having p-value <0.05 and q-value < 0.05 to limit the false discovery rate. Gene expression in the liver is highly influenced

by pattern of food intake. In the CR-2h groups, hepatic rhythms are influenced by the 2-hr feeding pattern, which only RAIN may be able to detect as cycling, and such gene(s) would not be reported as "cycling" in our final list. Fold changes across the day were calculated by the ratio between the average of the maximum RPKM across each day and the average of the minimum RPKM across each day. Daily fold change ratio between aged and young mice were calculated for the cycling genes in either young or aged mice fed *ad libitum*.

Gene Ontology

Gene Ontology analysis was performed with g:Profiler (R package g:Profiler, (*105*)) for both gene groups identified by differential expression and circadian analysis, using expressed genes as a custom background. Curated lists of the terms were chosen for figures, selecting 10 nonredundant and representative GO terms of the most significant top 25 terms.

Statistical Analysis

All behavioral and food intake data were collected using ClockLab (wheel-running activity) and Chamber Control (feeder software) from Actimetrics, Inc. Raw data were analyzed using R packages. Additional plots were generated and analyzed using Prism 9 (GraphPad Software, Inc.) and R (ggplot2 package). Pairwise comparisons were made using t-tests or two-way ANOVA with post hoc analyses as specified in the figure legends. Tests with adjusted $p < 0.05$ were deemed statistically significant. Unless otherwise stated, all values are presented as mean ± SEM, with statistical results presented as: * $p < 0.05$, ** $p < 0.01$, and *** $p < 0.001$. Spearman correlations were used to compare dependence between two variables (non-parametric, without assuming normal distribution). Linear regressions were used to test whether the slope differs from 1 (unity) when comparing amplitudes in feeding X vs Y.

Survival analysis of 43 mice under AL and 34-36 mice from each of the CR feeding conditions were compared using Log-rank Mantel-Cox. Maximal lifespan was defined as the 10th percentile of mice still alive and Fischer's exact test was used to detect pairwise statistical differences (*106*).

A

Ingredient	2018 Teklad Diet: % by weight	% kcal	source	F0170: grain-based: % by weight	% kcal	source	F0075: purified: % by weight	% kcal	source
Protein	18.6%	24%	soybean, brewers yeast	21.3%	25%	soybean, brewers yeast, fish meal, casein	18.7%	21%	casein
Fat	6.2%	18%	soybean oil	3.8%	10%	soybean oil, porcine animal fat	5.6%	14%	corn oil
Carbohydrate	44.2 % available	58%	wheat, corn	54%	64%	wheat, corn, banana flakes, sucrose, fructose, dextrose, beet, whey	59%	65%	sucrose, dextrose, corn syrup
Fiber	2.5 % crude<br>14.7% Neutral Detergent	N/A	cellulose	4.0%	N/A	cellulose	4.7%	N/A	cellulose
Energy Density	3.1 Kcal/g			3.35 Kcal/g			3.6 Kcal/g		
Pellet Size	Variable			Round: 300 mg (0.3" diameter)			Round: 300 mg (0.3" diameter)		
Detailed ingredients	Ground wheat, ground corn, wheat middlings, dehulled soybean meal, corn gluten meal, soybean oil, calcium carbonate, dicalcium phosphate, brewers dried yeast, iodized salt, Llysine, DL-methionine, choline chloride, kaolin, magnesium oxide, vitamin E, acetate, menadione sodium bisulfite complex (source of vitamin K activity), manganous oxide, ferrous sulfate, zinc oxide, niacin, calcium pantothenate, copper sulfate, pyridoxine hydrochloride, riboflavin, thiamin mononitrate, vitamin A acetate, calcium iodate, vitamin B12 supplement, folic acid, biotin,vitamin D3 supplement, cobalt carbonate			Ground Corn, Banana Flakes, Dehulled Soybean Meal, Corn Gluten Meal, Ground Wheat, Corn Gluten Feed, Fish Meal, Dehydrated Alfalfa Meal, Casein, Dried Whey, Sucrose, Fructose, Dextrose, Dried Beet Pulp, Soybean Oil, Porcine Animal Fat (preserved with BHA), Dried Brewers Yeast, Mineral Mix, Vitamin Mix, Magnesium Stearate, DL-Methionine, Choline Chloride, Sodium Propionate, Ascorbic Acid, Kaolin			Sucrose. Dextrose, Casein, Corn Oil, Mineral Mix, Cellulose, Corn Syrup, Calcium Silicate, Vitamin Mix, Magnesium Stearate, Choline Bitartrate, DL-Methionine, L-Cystine, Ascorbic Acid, Vitamin E Acetate, tBHQ		

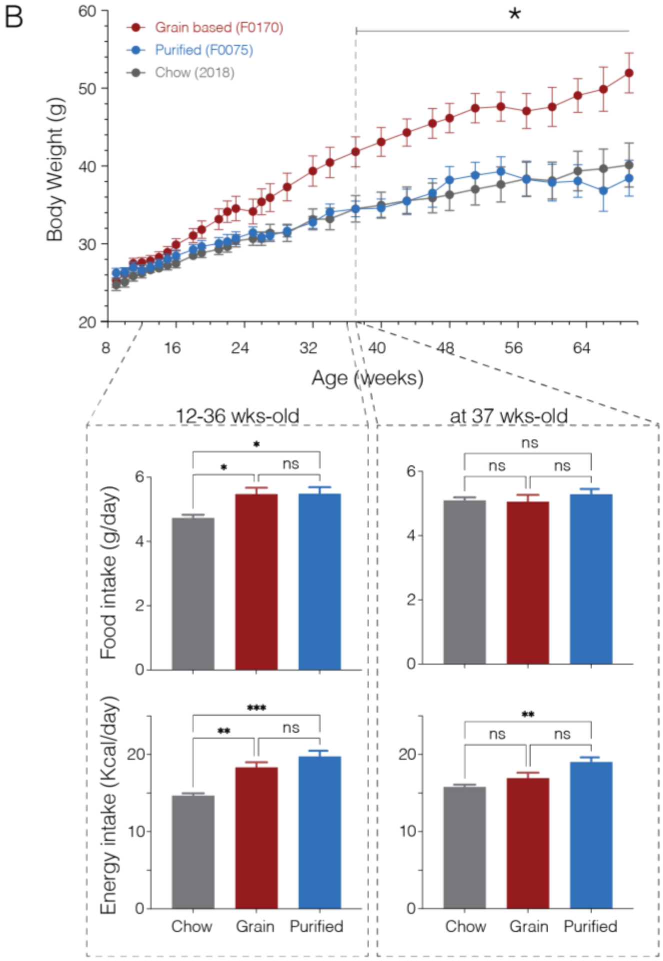

Fig. S1. Food selection for long-term experiments. A. Nutritional composition for standard lab chow (Teklad Global 2018, Envigo) and two types of 300 mg precision pellets, grain-based (F0170, Bio-Serv) and purified diet (F0075, Bio-Serv). **B.** Body weight gain (top), food intake and energy intake (bottom) for *ad libitum* mice fed with three different diets (Mean $\pm$ SEM, n = 9 per diet) from 12-36 weeks of age (left) and at 37 weeks-old (right). Asterisks in the bar plot represent significant ($p < 0.05$) results of one-way ANOVA with post-hoc comparisons.

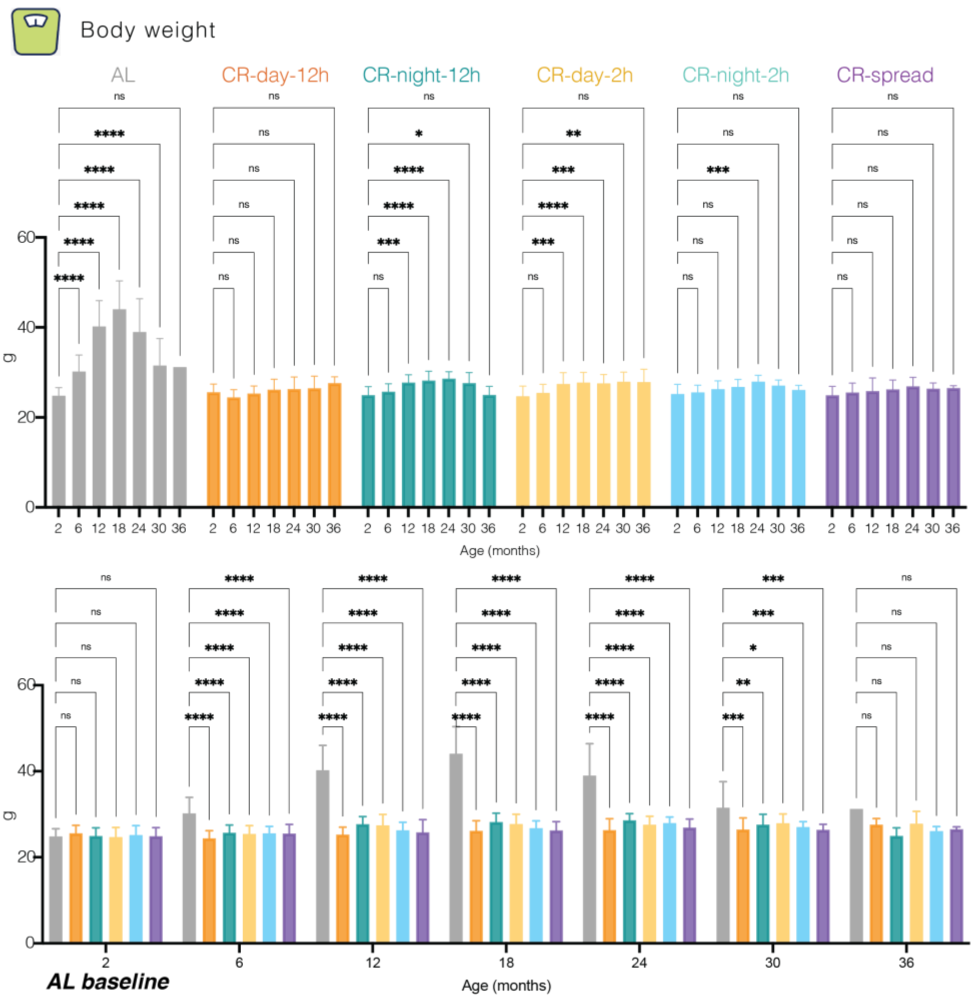

Source of Variation	% of total variation	P value	P value summary	Significant?
Interaction	21.86	<0.0001	****	Yes
Age	11.64	<0.0001	****	Yes
Feeding	7.320	<0.0001	****	Yes

ANOVA table	SS (Type III)	DF	MS	F (DFn, DFd)	P value
Interaction	7071	30	235.7	F (30, 1089) = 29.20	P<0.0001
Age	3766	6	627.6	F (6, 1089) = 77.77	P<0.0001
Feeding	2368	5	473.6	F (5, 1089) = 58.68	P<0.0001

Fig. S2. Body weight change across lifespan for 6 feeding conditions. Body weight was measured every 21 days but for clarity are plotted at 2 months and every 6 months thereafter. Body weights were measured in the morning (body weights change over the 24 hour cycle with higher weights reflecting recently consumed food – see Fig. S4). Gray, AL; orange, CR-day-12h; green, CR-night-12h, yellow, CR-day-2h; blue, CR-night-2h; purple, CR-spread. Data represents mean and SD, starting with N = 43 for AL and n = 34-36 for each of the CR conditions. Two-way ANOVA statistical test results are shown at the bottom.

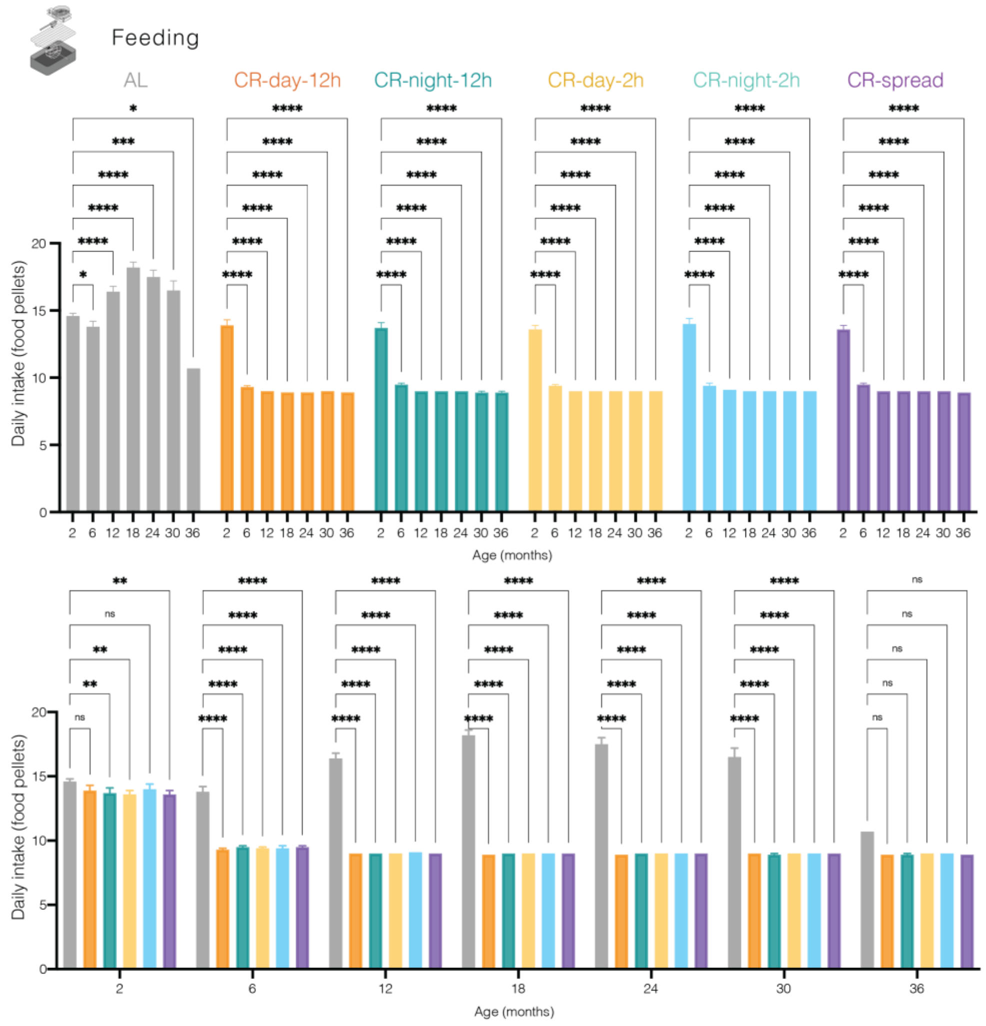

Source of Variation	% of total variation	P value	P value summary	Significant?
Interaction	14.56	<0.0001	****	Yes
Age	21.19	<0.0001	****	Yes
Feeding	11.57	<0.0001	****	Yes

ANOVA table	SS (Type III)	DF	MS	F (DFn, DFd)	P value
Interaction	1461	30	48.71	F (30, 1005) = 31.12	P<0.0001
Age	2126	6	354.4	F (6, 1005) = 226.4	P<0.0001
Feeding	1161	5	232.1	F (5, 1005) = 148.3	P<0.0001

Fig. S3. Feeding intake across lifespan for 6 feeding conditions. Y-axis indicates number of pellets taken per day (average of 21 days per mouse at each age). Data represents mean and SD, starting with N = 43 for AL and n = 34-36 for each of the CR conditions. Two-way ANOVA statistical test results are shown at the bottom.

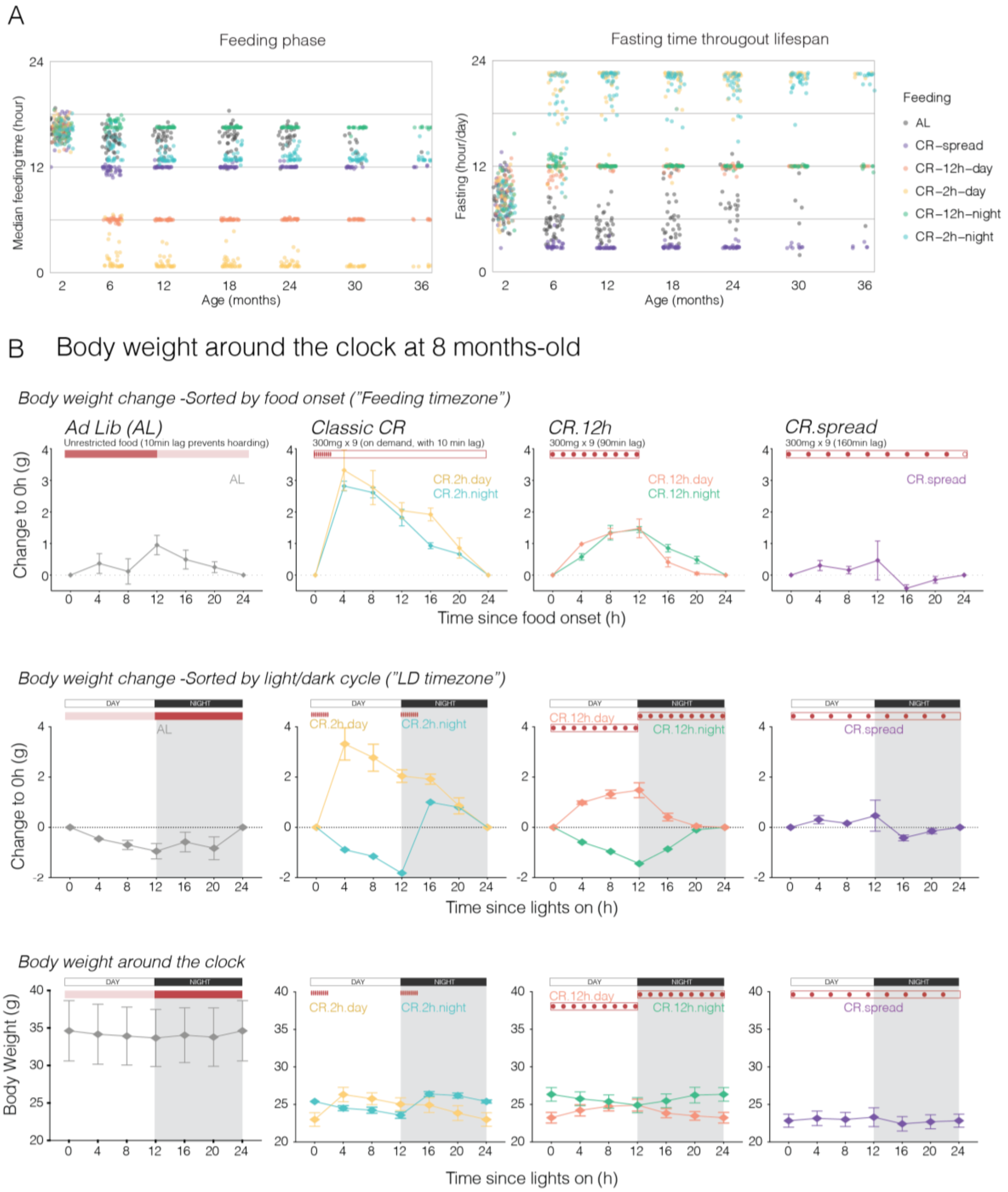

Fig. S4. A. Feeding phase as defined as time at which mice ate 50% of their daily allotment (left) and fasting duration (right) for each mouse in the longevity study, color-coded by feeding condition. Mean ± SEM, n = 34-43 animals per age per feeding condition (number of mice decline with age). **B.** Fluctuation of body weight across the day for each feeding condition at 8 months old. Mean ± SEM, n = 4 animals per time point per feeding condition.

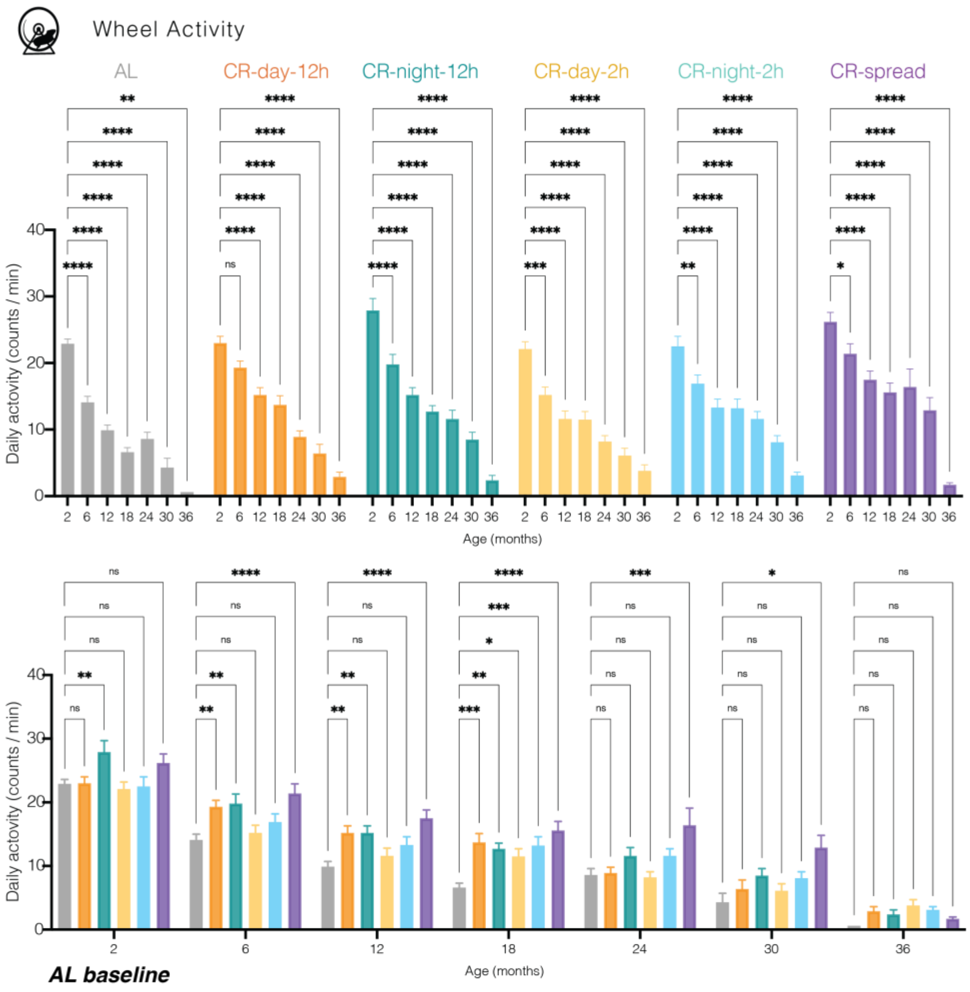

Source of Variation	% of total variation	P value	P value summary	Significant?
Interaction	1.808	0.2914	ns	No
Age	34.14	<0.0001	****	Yes
Feeding	1.985	<0.0001	****	Yes

ANOVA table	SS (Type III)	DF	MS	F (DFn, DFd)	P value
Interaction	1581	30	52.69	F (30, 1033) = 1.128	P=0.2914
Age	29852	6	4975	F (6, 1033) = 106.5	P<0.0001
Feeding	1735	5	347.0	F (5, 1033) = 7.427	P<0.0001

Fig. S5. Wheel Activity across lifespan for 6 feeding conditions. The Y-axis is the average total daily activity (counts/min, average of 21 days per mouse at each age). Data represents mean and SD, starting with N = 43 for AL and n = 34-36 for each of the CR conditions. Two-way ANOVA statistical test results are shown at the bottom.

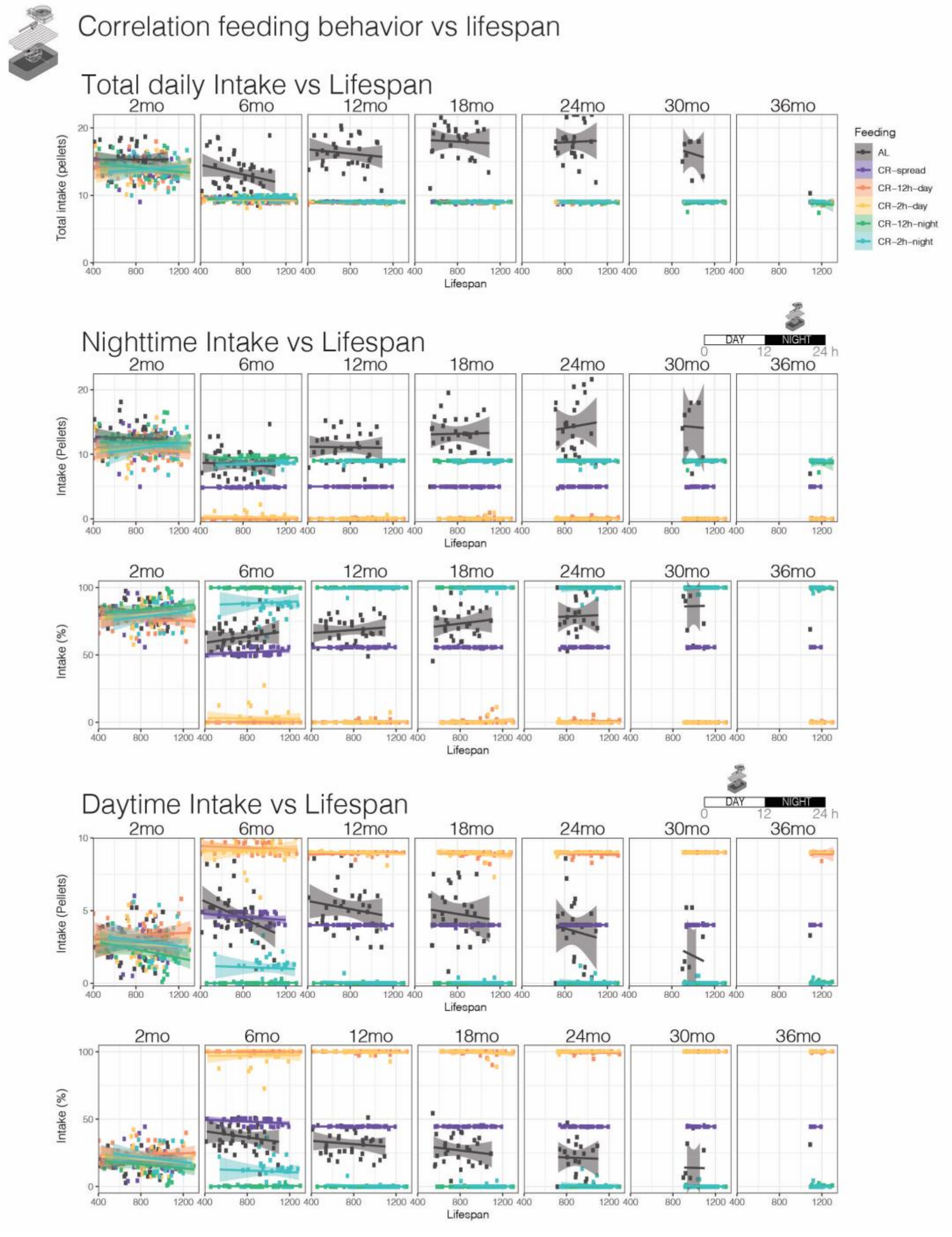

Fig. S6. Correlation plots for food intake across lifespan. Number of pellets consumed (averaged per mouse every 21 days) for 24h (top) or during either the nighttime (middle) or daytime (bottom) are represented. Lower panels in nighttime and daytime sets show percentage of pellets consumed in either nighttime or daytime from the 24h day. Each dot represents average data from one mouse. Regression lines are displayed and shaded areas represent SEM. n = 43 AL mice, n = 34-36 for CR groups at the beginning of the experiment.

Correlation body weight vs lifespan

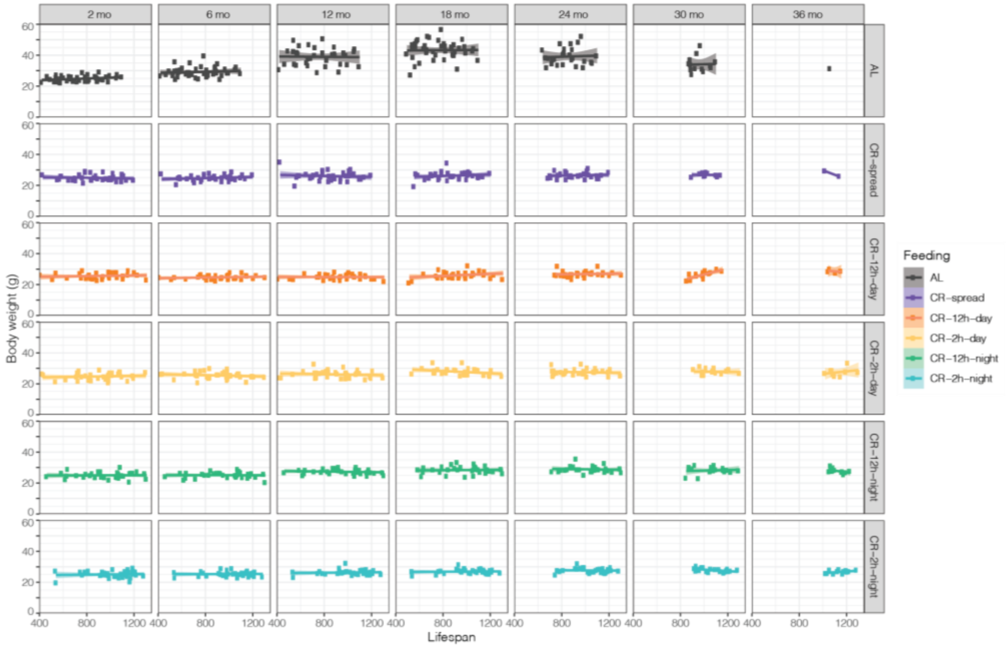

Fig. S7. Correlation of body weight and lifespan for 6 feeding conditions. Each dot represents data from one mouse. Regression lines are displayed and shaded areas represent SEM. n = 43 AL mice, n = 34-36 for CR groups at the beginning of the experiment.

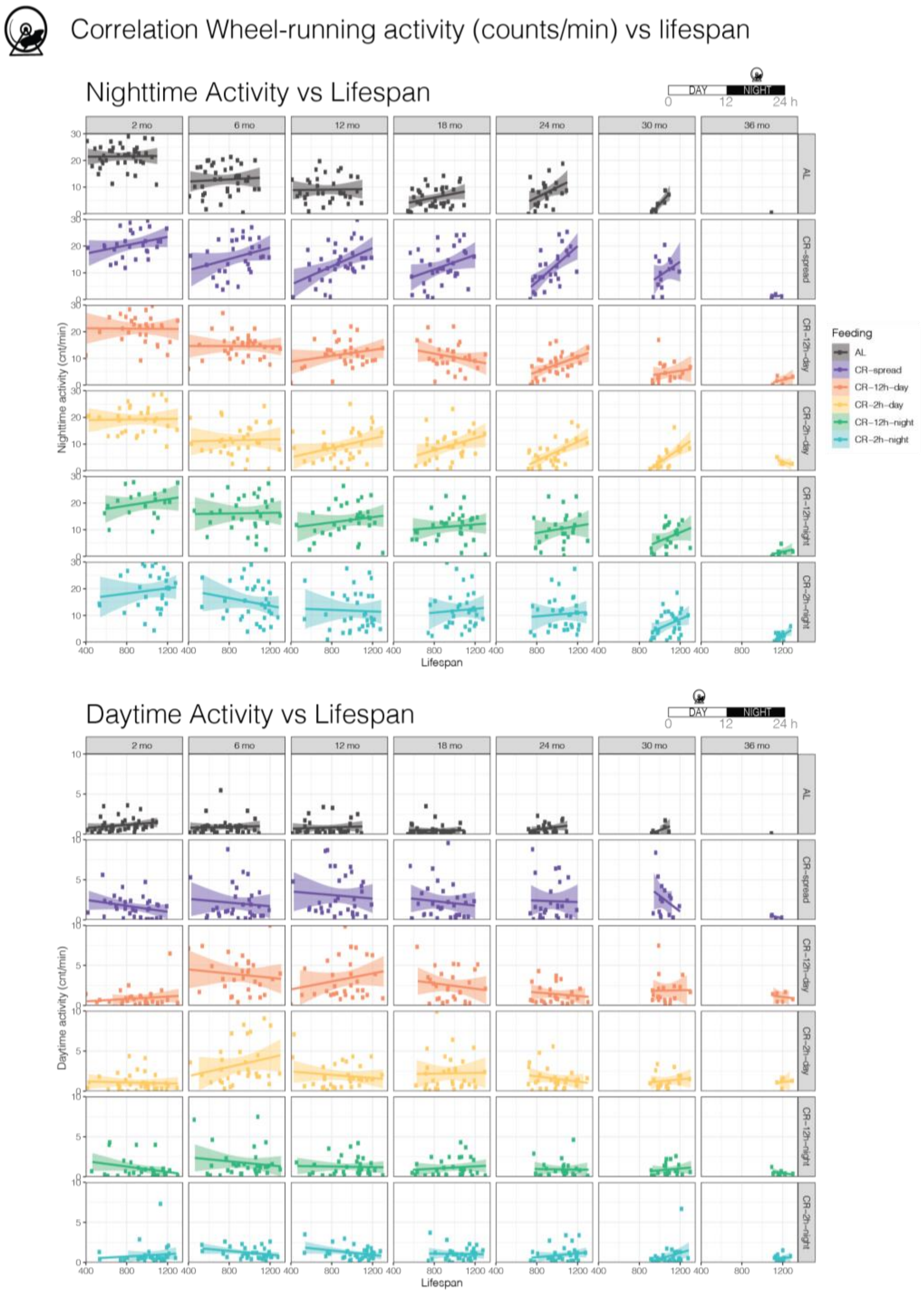

Fig. S8. Correlation of wheel running activity with lifespan for 6 feeding conditions. Top panel represents the nighttime activity (from lights off to lights on), and the bottom panel represents daytime activity (activity occurring from lights on to lights off). Each dot represents average data of 21 days from one mouse. Regression lines are displayed and shaded areas represent SEM. n = 43 AL mice, n = 34-36 for CR groups at the beginning of the experiment.

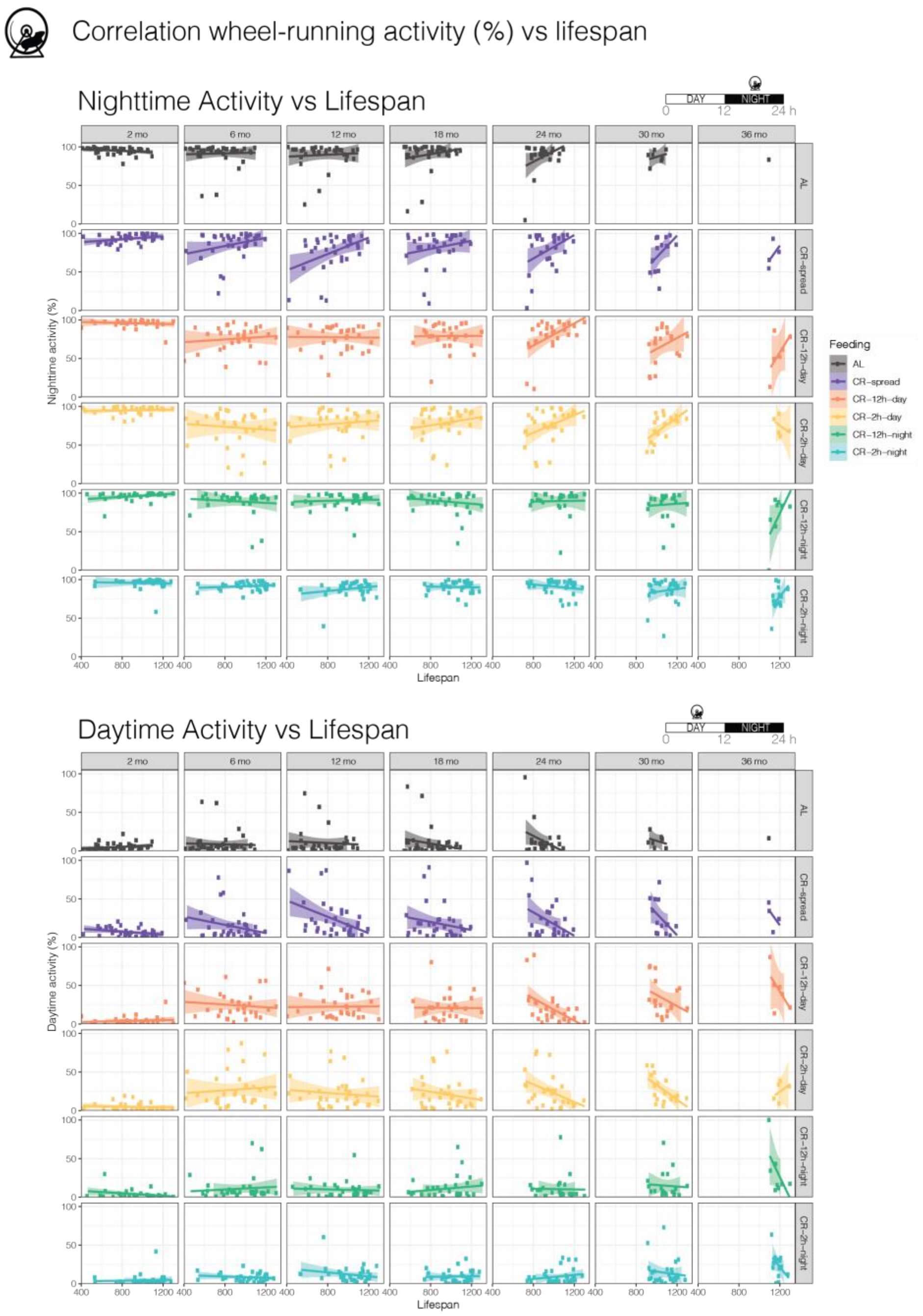

Fig. S9. Correlation of wheel running activity as a percentage of lifespan for 6 feeding conditions. Top panel represents the nighttime activity (from lights off to lights on), and the bottom panel represents daytime activity (activity occurring from lights on to lights off). Each dot represents average data of 21 days from one mouse. Regression lines are displayed and shaded areas represent SEM. n = 43 AL mice, n = 34-36 for CR groups at the beginning of the experiment.

A

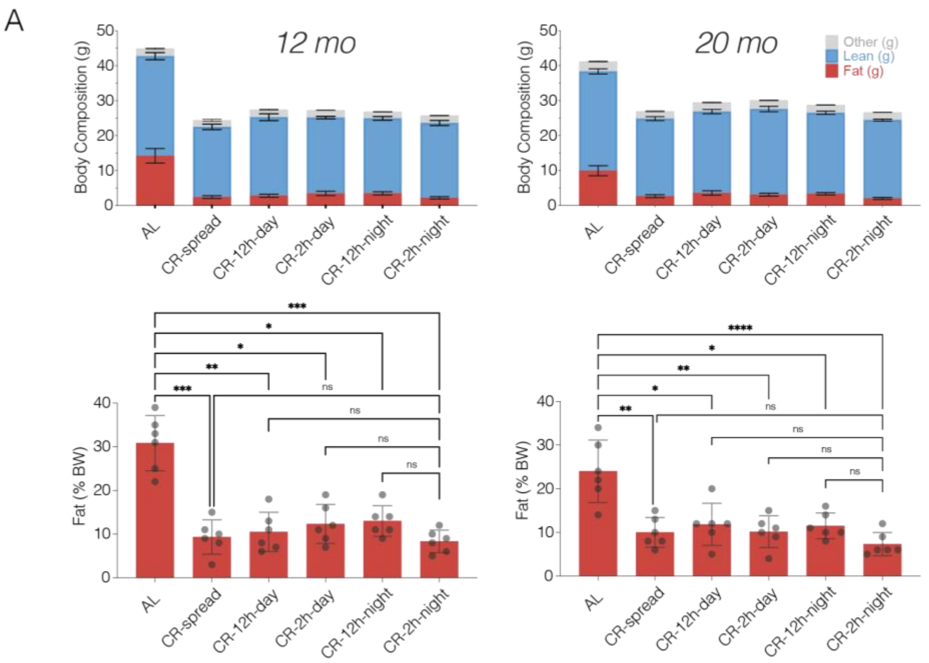

Feeding Group	12 months-old Mean rank diff.	Significant?	Summary	p-value	20 months-old Mean rank diff.	Significant?	Summary	p-value
AL vs. CR-spread	20.33	Yes	***	0.0008	17.33	Yes	**	0.0042
AL vs. CR-12h-day	19.17	Yes	**	0.0016	13.5	Yes	*	0.0259
AL vs. CR-2h-day	14.67	Yes	*	0.0156	16.58	Yes	**	0.0062
AL vs. CR-12h-night	12.5	Yes	*	0.0394	13.67	Yes	*	0.0241
AL vs. CR-2h-night	23.33	Yes	***	0.0001	23.92	Yes	****	<0.0001
CR-spread vs. CR-12h-day	-1.167	No	ns	0.8475	-3.833	No	ns	0.527
CR-spread vs. CR-2h-day	-5.667	No	ns	0.3503	-0.75	No	ns	0.9015
CR-spread vs. CR-12h-night	-7.833	No	ns	0.1967	-3.667	No	ns	0.5451
CR-spread vs. CR-2h-night	3	No	ns	0.621	6.583	No	ns	0.2773
CR-12h-day vs. CR-2h-day	-4.5	No	ns	0.4583	3.083	No	ns	0.6108
CR-12h-day vs. CR-12h-night	-6.667	No	ns	0.2718	0.1667	No	ns	0.9781
CR-12h-day vs. CR-2h-night	4.167	No	ns	0.4922	10.42	No	ns	0.0856
CR-2h-day vs. CR-12h-night	-2.167	No	ns	0.721	-2.917	No	ns	0.6303
CR-2h-day vs. CR-2h-night	8.667	No	ns	0.1532	7.333	No	ns	0.2262
CR-12h-night vs. CR-2h-night	10.83	No	ns	0.0742	10.25	No	ns	0.0907

B

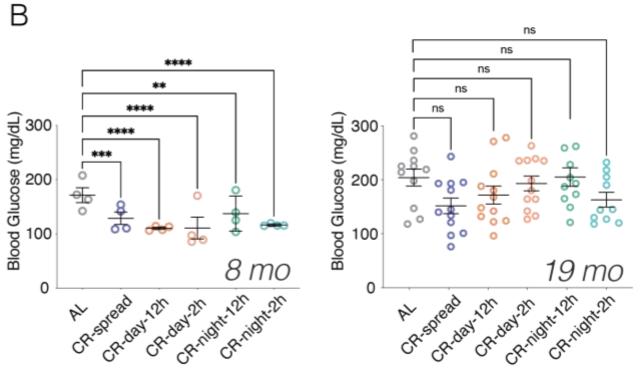

	Mean Diff.	Summary	Adj p-val
8mo			
AL vs. CR-spread	42.71	***	0.0004
AL vs. CR-day-12h	60.95	****	<0.0001
AL vs. CR-day-2h	65.68	****	<0.0001
AL vs. CR-night-12h	37.78	**	0.0026
AL vs. CR-night-2h	55.04	****	<0.0001
19mo			
AL vs. CR-spread	18.55	ns	0.1231
AL vs. CR-day-12h	12.63	ns	0.6297
AL vs. CR-day-2h	3.379	ns	>0.9999
AL vs. CR-night-12h	0.8182	ns	>0.9999
AL vs. CR-night-2h	15.75	ns	0.3417

Fig. S10. Body composition and blood glucose at two different ages for all feeding conditions. A. Body composition is shown in grams (top) and as percent of body weight that is fat (bottom). Because body weight changes daily after the feeding events (fig. S4B), the values here represent averaged data from measurements taken at ZT0 and ZT12 (n = 6 mice/group). Mean ± SEM, each mouse represented as a point, n = 6 mice per condition. ANOVA statistical test results are shown at the bottom. B. Blood glucose levels (plasma) at two ages. Mean ± SEM, n =4 at 8 months-old (averaged of 6 timepoints) and n =12 for 19 months-old. ANOVA statistical test results with multiple comparison are shown at the right.

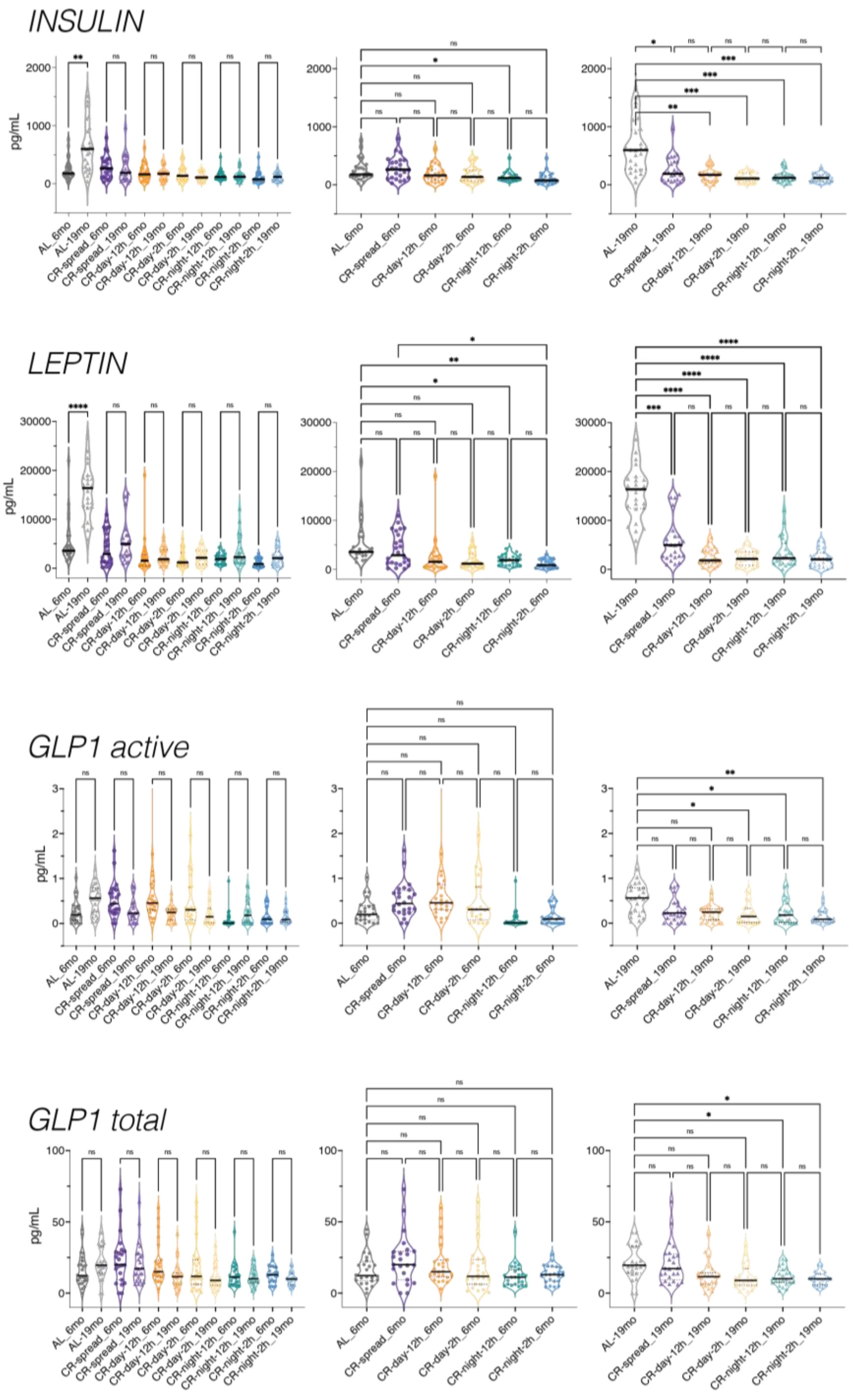

Fig. S11. Plasma hormone levels across age and feeding conditions. Samples were taken every 4h for 48h in the mice used for gene expression experiments. Samples were then graphed by pooling all 24 samples together (n = 24 mice per age per feeding condition). Violin plots are shown with each mouse represented as a point and the median as a black line.

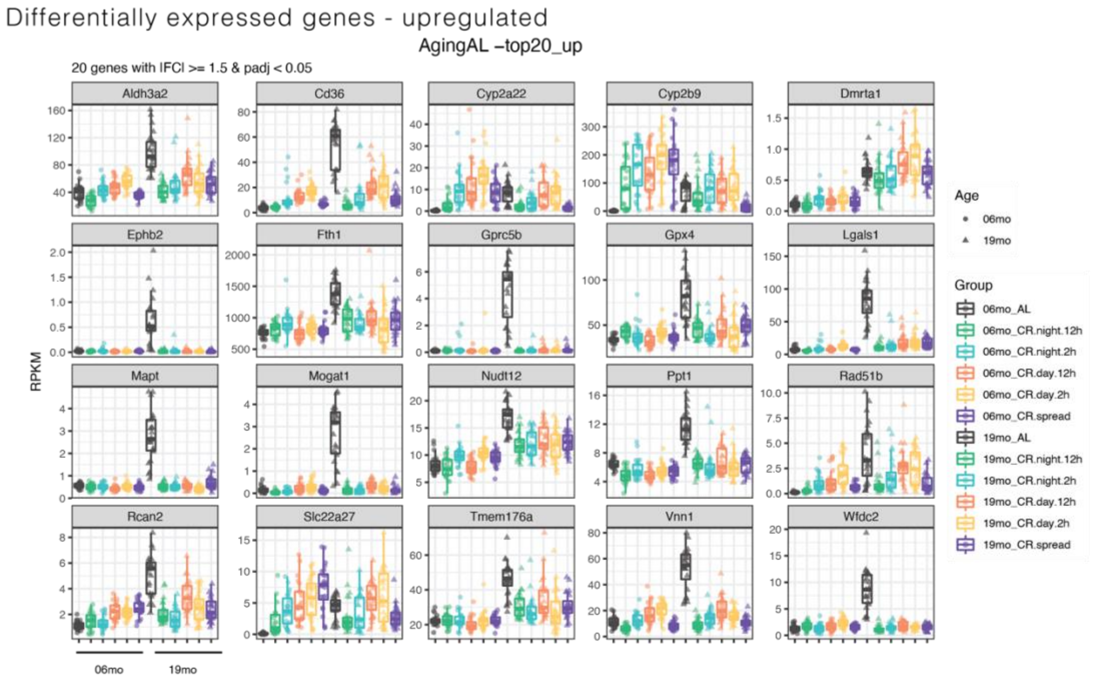

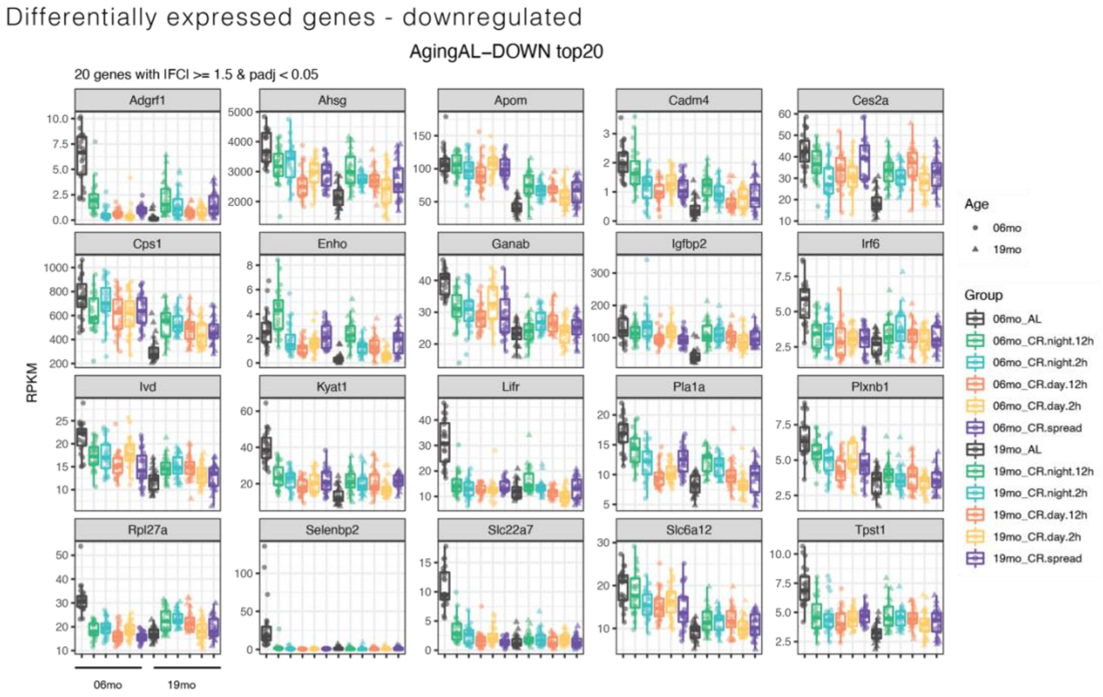

Fig. S12. Top 20 genes whose expression changed with age under AL. Top panel represents the 20 most significant genes that are upregulated with age. Bottom panel represents the 20 most significant genes that are downregulated with age. Liver gene expression data from n = 24 mice per age per feeding condition. Box plots show the data with each mouse represented as a point. Criteria for significance is change with adjusted p value <0.05 (FDR) and a fold change of 1.5 or more. For all genes the CR gene expression levels at both 6 and 19 months are also shown.

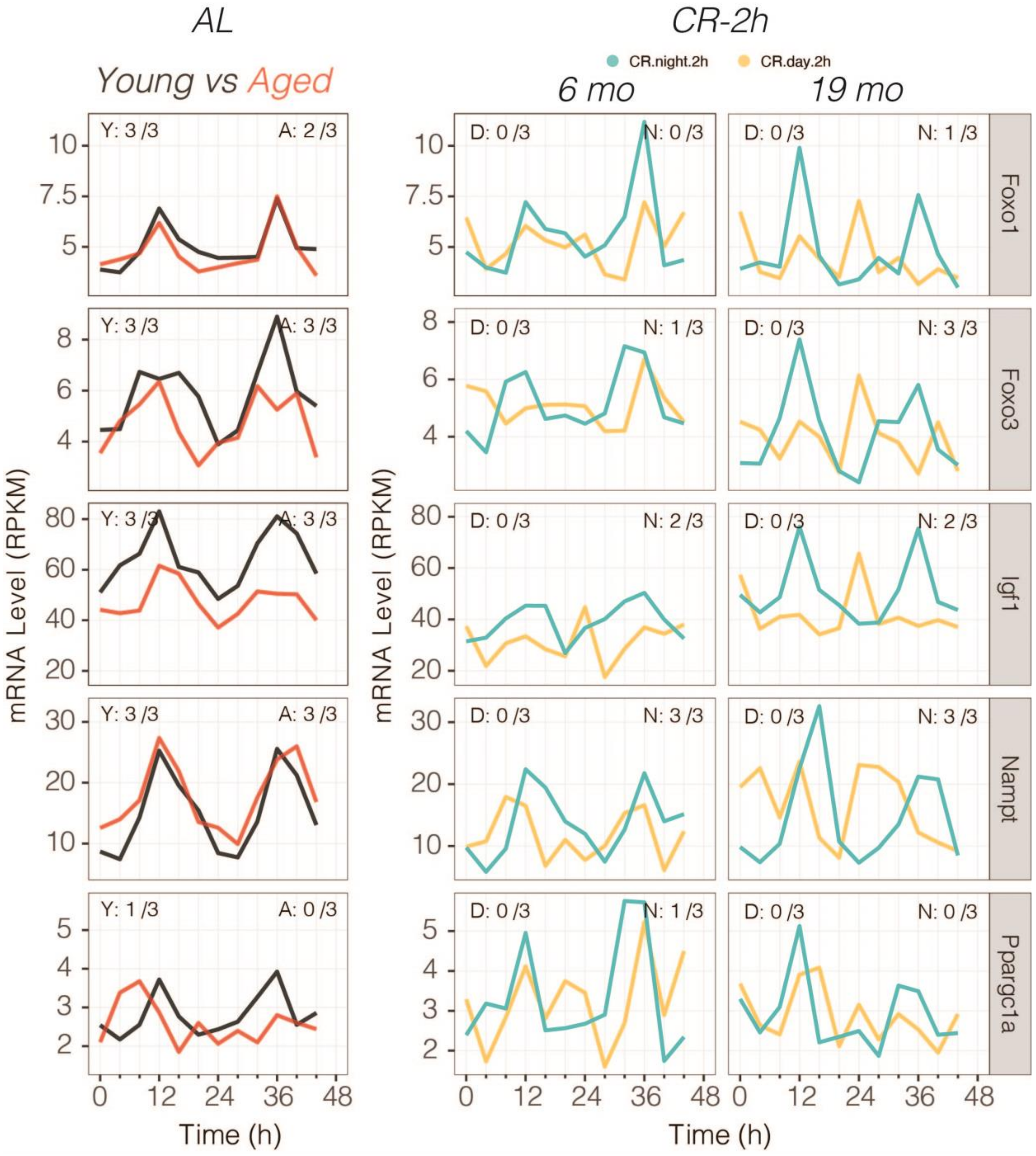

Fig. S13. Examples of circadian profiles of pro-aging and pro-longevity genes in liver. Expression profiles for young (6 months-old, dark grey) and aged (19 month-old, red) in the control group AL (left, related to Fig 5). Expression profiles are shown comparing CR-night-2h (blue) and CR-day-2h (yellow) for young (middle) and aged (right) mice (related to Fig. 6). Numbers on the bottom right on each plot represent the number of algorithms that meet the criteria for circadian expression (p and $q < 0.05$) out of the 3 circadian algorithms tested (ARSER, JTK and RAIN). Abbreviations: young (Y), aged (A), N (night) and D (day).

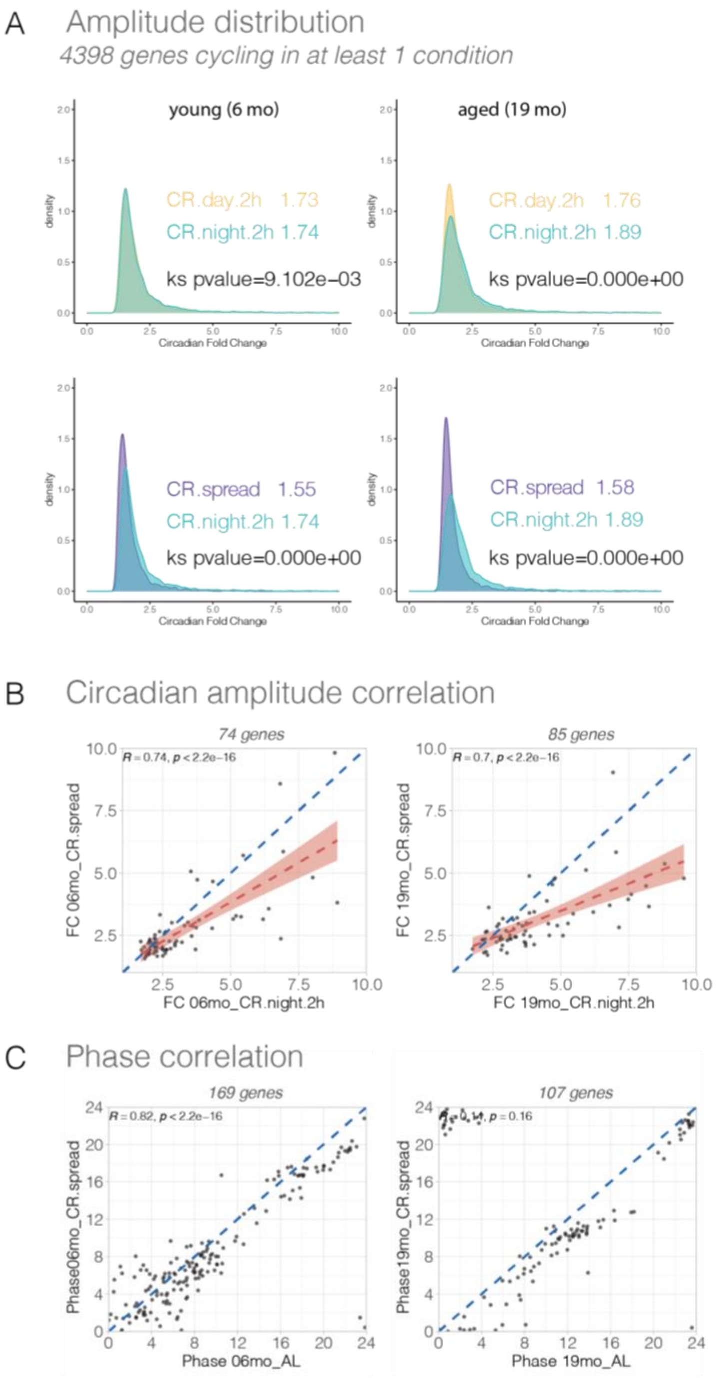

Fig. S14. Circadian analysis of gene expression from liver. **A.** Amplitude distribution of genes identified as cycling in at least one feeding condition. Values reported are the circadian fold change. Kolmogorov Smirnov, KS statistical test. **B.** Correlation of circadian fold change between CR-night-2h and CR-spread at two ages, for genes that cycle in both conditions. Dashed red line is the linear regression with shaded area representing the SEM. **C.** Phase correlation (time of maximum expression for a cycling gene) at two different ages between CR-spread and ad libitum for the genes that cycle in both conditions.

Data S1. (separate file)

Mouse activity, maximum lifespan and histopathology results under 6 feeding paradigms. Wheel-running and feeding activity throughout lifespan per group for all 6 conditions tested. Averaged data by epoch (every 21 days, when cage change occurs). Epoch 1 starts at 2 months of age, when mice were installed. Both epoch 1 and 2 represent AL baseline for all 6 groups (1 tab). Statistical comparison of maximum lifespan using Fischer's Exact test, results from n=34-43 mice per condition, 6 feeding conditions (1 tab). Blinded histopathology analysis of tissues collected at death after necropsy. Results from n=24-36 mice per condition, 6 feeding conditions (1 tab). Related to Fig. 2.

Data S2. (separate file)

Age-related differential gene expression results in mouse liver. Differentially expressed gene lists and results from young (6 months-old) vs aged (19 months-old) mice, DEseq2. Liver gene expression data from n = 24 mice per age per feeding condition, 6 feeding conditions. Related to Fig. 3-4.

Data S3. (separate file)

Raw count data for liver mRNA expression. Data from n = 24 mice per age per feeding condition. Young (6 month-old) and aged (19 month-old) mice from 6 feeding conditions, biological duplicates, collected in the dark every 4 hours for two days. Data used for Deseq2 analysis. Related to Fig. 3-4.

Data S4. (separate file)

Gene Ontology analysis for differentially expressed and circadian genes in mouse liver. Results for both DESeq2 (6 tabs) and circadian gene expression (2 tabs). Related to Fig. 3-6.

Data S5. (separate file)

Normalized counts data for liver mRNA expression. RPKM (Reads Per Kilobase per million) liver gene expression data from n = 24 mice per age per feeding condition. Young (6 months-old) and aged (19 months-old) mice from 6 feeding conditions, biological duplicates, collected in the dark every 4 hours for two days. Data used for the circadian analysis. Related to Fig. 5-6.

Data S6. (separate file)

Circadian analysis results for mouse liver. Liver gene expression data (mRNA-seq) from n = 24 mice per age per feeding condition. 6 feeding conditions, biological duplicates, collected in the dark every 4 hours for two days. Results from circadian algorithms ARSER, JTK_CYCLE and RAIN. Related to Fig. 5-6.
